# Scaling Equilibrium Propagation to Deep ConvNets by Drastically Reducing Its Gradient Estimator Bias

**DOI:** 10.3389/fnins.2021.633674

**Published:** 2021-02-18

**Authors:** Axel Laborieux, Maxence Ernoult, Benjamin Scellier, Yoshua Bengio, Julie Grollier, Damien Querlioz

**Affiliations:** ^1^Université Paris-Saclay, CNRS, Centre de Nanosciences et de Nanotechnologies, Palaiseau, France; ^2^Unité Mixte de Physique, CNRS, Thales, Université Paris-Saclay, Palaiseau, France; ^3^Mila, Université de Montréal, Montreal, QC, Canada; ^4^Canadian Institute for Advanced Research, Toronto, ON, Canada

**Keywords:** equilibrium propagation, energy based models, biologically plausible deep learning, neuromorphic computing, on-chip learning, deep convolutional neural network, learning algorithms

## Abstract

Equilibrium Propagation is a biologically-inspired algorithm that trains convergent recurrent neural networks with a local learning rule. This approach constitutes a major lead to allow learning-capable neuromophic systems and comes with strong theoretical guarantees. Equilibrium propagation operates in two phases, during which the network is let to evolve freely and then “nudged” toward a target; the weights of the network are then updated based solely on the states of the neurons that they connect. The weight updates of Equilibrium Propagation have been shown mathematically to approach those provided by Backpropagation Through Time (BPTT), the mainstream approach to train recurrent neural networks, when nudging is performed with infinitely small strength. In practice, however, the standard implementation of Equilibrium Propagation does not scale to visual tasks harder than MNIST. In this work, we show that a bias in the gradient estimate of equilibrium propagation, inherent in the use of finite nudging, is responsible for this phenomenon and that canceling it allows training deep convolutional neural networks. We show that this bias can be greatly reduced by using symmetric nudging (a positive nudging and a negative one). We also generalize Equilibrium Propagation to the case of cross-entropy loss (by opposition to squared error). As a result of these advances, we are able to achieve a test error of 11.7% on CIFAR-10, which approaches the one achieved by BPTT and provides a major improvement with respect to the standard Equilibrium Propagation that gives 86% test error. We also apply these techniques to train an architecture with unidirectional forward and backward connections, yielding a 13.2% test error. These results highlight equilibrium propagation as a compelling biologically-plausible approach to compute error gradients in deep neuromorphic systems.

## 1. Introduction

How synapses in hierarchical neural circuits are adjusted throughout learning a task remains a challenging question called the credit assignment problem (Richards et al., 2019). Equilibrium Propagation (EP) (Scellier and Bengio, 2017) provides a biologically plausible solution to this problem in artificial neural networks. EP is an algorithm for convergent recurrent neural networks (RNNs) which, by definition, are given a static input and whose recurrent dynamics converge to a steady state corresponding to the prediction of the network. EP proceeds in two phases, bringing the network to a first steady state, then nudging the output layer of the network toward a ground-truth target until reaching a second steady state. During the second phase of EP, the perturbation originating from the output layer propagates forward in time to upstream layers, creating local error signals that match exactly those that are computed by Backpropagation Through Time (BPTT), the canonical approach for training RNNs (Ernoult et al., 2019). We refer to Scellier and Bengio (2019) for a comparison between EP and recurrent backpropagation (Almeida, 1987; Pineda, 1987). Owing to this strong theoretical guarantee, EP can provide leads for understanding biological learning (Lillicrap et al., 2020). Moreover, the spatial locality of the learning rule prescribed by EP and the possibility to make it also local in time (Ernoult et al., 2020) is highly attractive for designing energy-efficient neuromorphic hardware implementations of gradient-based learning algorithms (Ernoult et al., 2020; Foroushani et al., 2020; Ji and Gross, 2020; Kendall et al., 2020; Martin et al., 2020; Zoppo et al., 2020).

To meet these expectations, however, EP should be able to scale to complex tasks. Until now, works on EP (Scellier and Bengio, 2017; O'Connor et al., 2018, 2019; Ernoult et al., 2019, 2020) limited their experiments to the MNIST classification task and shallow network architectures. Despite the theoretical guarantees of EP, the literature suggests that no implementation of EP has thus far succeeded to match the performance of standard deep learning approaches to train deep networks on hard visual tasks. This problem is even more challenging when using a more bio-plausible topology where the synaptic connections of the network are unidirectional: existing proposals of EP in this situation (Scellier et al., 2018; Ernoult et al., 2020) lead to a degradation of accuracy on MNIST compared to standard EP. In this work, we show that performing the second phase of EP with nudging strength of constant sign induces a systematic first order bias in the EP gradient estimate which, once canceled, unlocks the training of deep convolutional neural networks (ConvNets), with bidirectional or unidirectional connections and with performance closely matching that of BPTT on CIFAR-10. We also propose to implement the neural network predictor as an external softmax readout. This modification preserves the local nature of EP and allows us to use the cross-entropy loss, contrary to previous approaches using the squared error loss, and where the predictor takes part in the free dynamics of the system.

Other biologically plausible alternatives to backpropagation (BP) have attempted to scale to hard vision tasks. Bartunov et al. (2018) investigated the use of feedback alignment (Lillicrap et al., 2016) and variants of target propagation (Lecun, 1987; Bengio, 2014) on CIFAR-10 and ImageNet, showing that they perform significantly worse than backpropagation. When the alignment between forward and backward weights is enhanced with extra mechanisms (Akrout et al., 2019), feedback alignment performs better on ImageNet than sign-symmetry (Xiao et al., 2018), where feedback weights are taken to be the sign of the forward weights, and almost as well as backpropagation. However, in feedback alignment and target propagation, the error feedback does not affect the forward neural activity and is instead routed through a distinct backward pathway, an issue that EP avoids. Payeur et al. (2020) proposed a burst-dependent learning rule that also addresses this problem and whose rate-based equivalent, relying on the use of specialized synapses and complex network topology, has been benchmarked against CIFAR-10 and ImageNet. Related works on implicit models (Bai et al., 2019) have shown that training deep networks can be framed as solving a fixed point (steady state) equation, leading to an analytical backward pass. This framework was shown to solve challenging vision tasks (Bai et al., 2020). While the use of a steady state is common with EP, the process to reach the steady state as well as the learning rule are different. In comparison with these approaches, EP offers a minimalistic circuit requirement to handle both inference and gradient computation, which makes it an outstanding candidate for energy-efficient neuromorphic learning hardware design.

More specifically, the contributions of this work are the following:

- We introduce a new method to estimate the gradient of the loss based on three steady states instead of two (section 3.1). This approach enables us to achieve 11.68% test error on CIFAR-10, with 0.6% performance degradation only with respect to BPTT. Conversely, we show that using a nudging strength of constant sign yields 86.64% test error.
- We propose to implement the output layer of the neural network as a softmax readout, which subsequently allows us to optimize the cross-entropy loss function with EP. This method improves the classification performance on CIFAR-10 with respect to the use of the squared error loss and is also closer to the one achieved with BPTT (section 3.2).
- Finally, based on ideas of Scellier et al. (2018) and Kolen and Pollack (1994), we adapt the learning rule of EP for architectures with distinct (unidirectional) forward and backward connections, yielding only 1.5% performance degradation on CIFAR-10 compared to bidirectional connections (section 2.4).

## 2. Background

### 2.1. Convergent RNNs With Static Input

We consider the setting of supervised learning where we are given an input *x* (e.g., an image) and want to predict a target *y* (e.g., the class label of that image). To solve this type of task, Equilibrium Propagation (EP) relies on convergent RNNs, where the input of the RNN at each time step is static and equal to *x*, and the state *s* of the neural network converges to a steady-state s_*_. EP applies to a wide class of convergent RNNs, where the transition function derives from a scalar primitive^1^ Φ (Ernoult et al., 2019). In this situation, the dynamics of a network with parameters θ, usually synaptic weights, is given by

(1) $$\begin{array}{l} s_{t+1}=\frac{\partial \Phi }{\partial s}\left(x,s_{t},\theta \right), \end{array}$$

where *s*_*t*_ is the state of the RNN at time step *t*. After the dynamics have converged at some time step *T*, the network reaches the steady state *s*_*T*_ = s_*_, which, by definition, satisfies:

(2) $$\begin{array}{l} s_{*}=\frac{\partial \Phi }{\partial s}\left(x,s_{*},\theta \right). \end{array}$$

Formally, the goal of learning is to optimize θ to minimize the loss at the steady state $L^{*}=\ell \left(s_{*},y\right)$, where ℓ is a differentiable cost function. While we did not investigate theoretical guarantees ensuring the convergence of the dynamics, we refer the reader to Scarselli et al. (2008) for sufficient conditions on the transition function to ensure convergence. In practice, we always observe the convergence to a steady-state.

### 2.2. Training Procedures for Convergent RNNs

#### 2.2.1. Equilibrium Propagation (EP)

Scellier and Bengio (2017) introduced Equilibrium Propagation in the case of real time dynamics. Subsequent work adapted it to discrete-time dynamics, bringing it closer to conventional deep learning (Ernoult et al., 2019). EP consists of two distinct phases. During the first (“free”) phase, the RNN evolves according to Equation (1) for *T* time steps to ensure convergence to a first steady state s_*_. During the second (“nudged”) phase of EP, a nudging term $-\beta \frac{\partial \ell }{\partial s}$ is added to the dynamics, with β a small scaling factor. Denoting $s_{0}^{\beta }$, $s_{1}^{\beta }$, $s_{2}^{\beta }$... the states during the second phase, the dynamics reads

(3) $$\begin{array}{l} s_{0}^{\beta }=s_{*},\text{ and }\forall t>0,\;s_{t+1}^{\beta }=\frac{\partial \Phi }{\partial s}\left(x,s_{t}^{\beta },\theta \right)-\beta \frac{\partial \ell }{\partial s}\left(s_{t}^{\beta },y\right). \end{array}$$

The RNN then reaches a new steady state denoted $s_{*}^{\beta }$. Scellier and Bengio (2017) proposed the EP learning rule, denoting η the learning rate applied:

(4) $$\begin{array}{l} \;\Delta \theta =\eta {\hat{\nabla }}^{\text{EP}}\left(\beta \right),\text{ where}\\ \begin{array}{l} {\hat{\nabla }}^{\text{EP}}\left(\beta \right)\overset{\Delta }{=}\frac{1}{\beta }\left(\frac{\partial \Phi }{\partial \theta }\left(x,s_{*}^{\beta },\theta \right)-\frac{\partial \Phi }{\partial \theta }\left(x,s_{*},\theta \right)\right). \end{array} \end{array}$$

They proved that this learning rule performs stochastic gradient descent in the limit β → 0:

(5) $$\begin{array}{l} \underset{\beta \to 0}{\text{lim}}{\hat{\nabla }}^{\text{EP}}\left(\beta \right)=-\frac{\partial L^{*}}{\partial \theta }. \end{array}$$

#### 2.2.2. Equivalence of Equilibrium Propagation and Backpropagation Through Time (BPTT)

The convergent RNNs considered by EP can also be trained by Backpropagation Through Time (BPTT). At each BPTT training iteration, the first phase is performed for *T* time steps until the network reaches the steady state *s*_*T*_ = s_*_. The loss at the final time step is computed and the gradients are subsequently backpropagated through the computational graph of the first phase, backward in time.

Let us denote ∇^BPTT^(*t*) the gradient computed by BPTT truncated to the last *t* time steps (*T* − *t*, …, *T*), which we define formally in Supplementary Material (section 1).

A theorem derived by Ernoult et al. (2019), inspired from Scellier and Bengio (2019), shows that, provided convergence in the first phase has been reached after *T* − *K* time steps (i.e., *s*_*T*−*K*_ = *s*_*T*−*K*+1_ = … = *s*_*T*_ = s_*_), the gradients of EP match those computed by BPTT in the limit β → 0, in the first *K* time steps of the second phase for fully connected and convolutional architectures including pooling operations:

(6) $$\begin{array}{l} \forall t=1,2,\ldots ,K,\\ {\hat{\nabla }}^{\text{EP}}\left(\beta ,t\right)\overset{\Delta }{=}\frac{1}{\beta }\left(\frac{\partial \Phi }{\partial \theta }\left(x,s_{t}^{\beta },\theta \right)-\frac{\partial \Phi }{\partial \theta }\left(x,s_{*},\theta \right)\right)\overset{\;}{\underset{\beta \to 0}{\to }}\nabla^{\text{BPTT}}\left(t\right). \end{array}$$

### 2.3. Convolutional Architectures for Convergent RNNs

**Figure 1:**
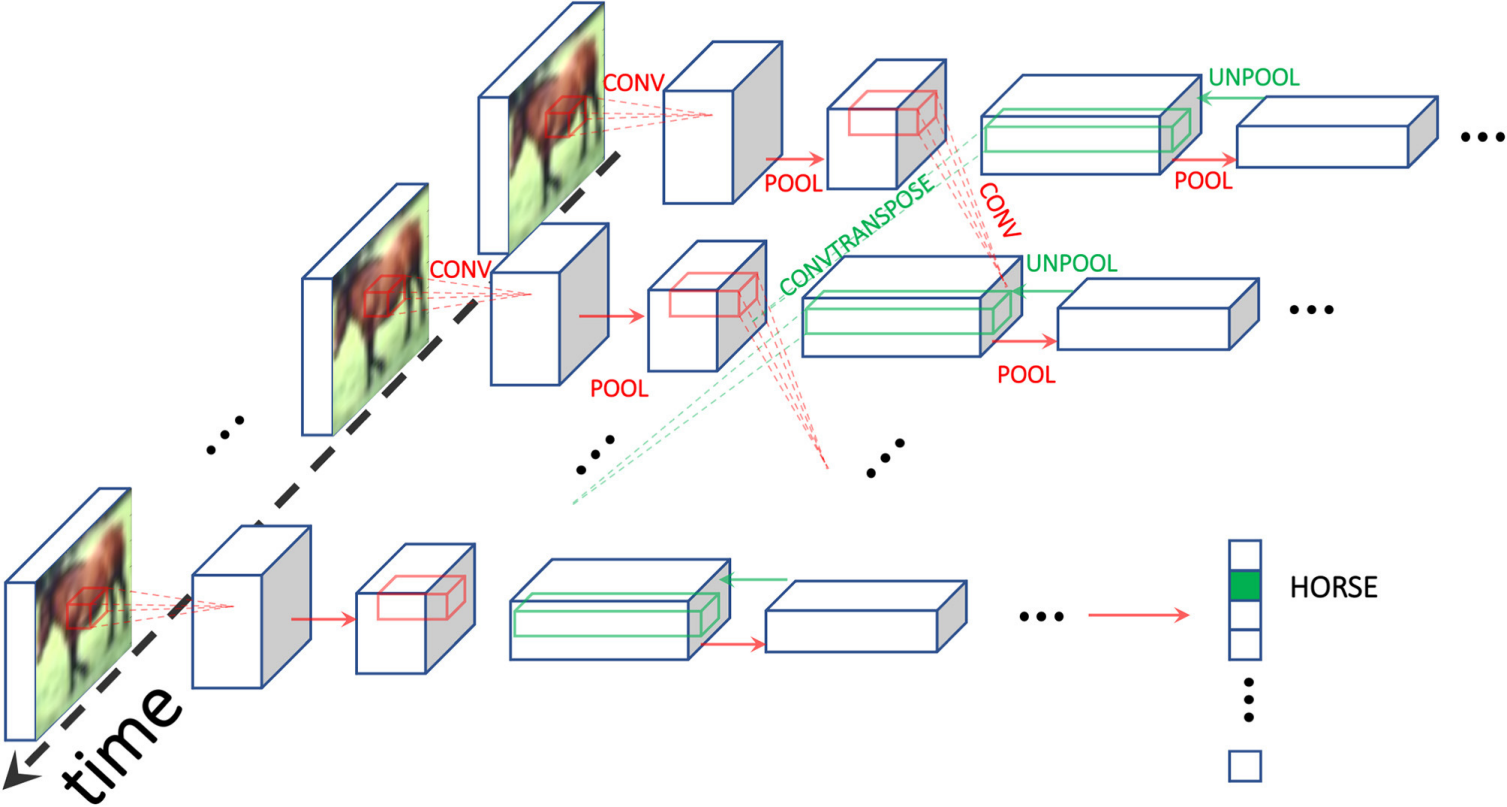
Schematic of the architecture used. We use Equilibrium Propagation (EP) to train a recurrent ConvNet receiving a static input. Red (resp. green) arrows depict forward (resp. backward) operations, with convolutions and transpose convolutions happening through time. At the final time step, the class prediction is carried out. The use of RNNs is inherent in the credit assignment of EP which uses of the temporal variations of the system as error signals for the gradient computation.

A convolutional architecture for convergent RNNs with static input was introduced by Ernoult et al. (2019) and successfully trained with EP on the MNIST dataset. In this architecture, presented in Figure 1, we define *N*^conv^ and *N*^fc^ the number of convolutional and fully connected layers respectively, and $N^{\text{tot}}\overset{\Delta }{=}N^{\text{conv}}+N^{\text{fc}}$. *w*_*n*+1_ denotes the weights connecting *s*^*n*^ to *s*^*n*+1^, with *s*_0_ = *x*. To simplify notations, we use distinct operators to differentiate whether *w*_*n*_ is a convolutional layer or a fully connected layer: respectively ⋆ for convolutions and · for linear layers. The primitive function can therefore be defined as:

(7) $$\begin{array}{l} \Phi \left(x,\left\{s^{n}\right\}\right)=\overset{N^{\text{conv}}-1}{\underset{n=0}{\sum }}s^{n+1}•P\left(w_{n+1}⋆s^{n}\right)\\ \begin{array}{l} \;+\overset{N^{\text{tot}}-1}{\underset{n=N^{\text{conv}}}{\sum }}s^{n+1⊤}\cdot w_{n+1}\cdot s^{n}, \end{array} \end{array}$$

where • is the Euclidean scalar product generalized to pairs of tensors with same arbitrary dimension, and P is a pooling operation. Combining Equations (1) and (7), and restricting the space of the state variables to [0, 1], yield the dynamics:

(8) $$\begin{array}{l} \{\begin{array}{c} s_{t+1}^{n}=\sigma \left(P\left(w_{n}⋆s_{t}^{n-1}\right)+{\tilde{w}}_{n+1}⋆P^{-1}\left(s_{t}^{n+1}\right)\right),1\leq n\leq N^{\text{conv}}\\ s_{t+1}^{n}=\sigma \left(w_{n}\cdot s_{t}^{n-1}+w_{n+1}^{⊤}\cdot s_{t}^{n+1}\right),N^{\text{conv}}<n<N^{\text{tot}} \end{array} \end{array}$$

where σ is an activation function bounded between 0 and 1. Transpose convolution and inverse pooling are respectively defined through the convolution by the flipped kernel $\tilde{w}$ and $P^{-1}$. Plugging Equation (7) into Equation (4) yields the local learning rule $\Delta \theta_{ij}=\eta \left(s_{i,*}^{\beta }s_{j,*}^{\beta }-s_{i,*}s_{j,*}\right)/\beta$ for a parameter θ_*ij*_ linking neurons *i* and *j*. Supplementary Material (section 4) provides the implementation details of this model.

### 2.4. Equilibrium Propagation With Unidirectional Synaptic Connections

We have seen that in the standard formulation of EP, the dynamics of the neural network derive from a function Φ (Equation 1) called the primitive function. This formulation implies the existence of bidirectional synaptic connections between neurons. For better biological plausibility, a more general formulation of EP circumvents this requirement and allows training networks with distinct (unidirectional) forward and backward connections (Scellier et al., 2018; Ernoult et al., 2020). This feature is also desirable for hardware implementations of EP. Although some analog implementations of EP naturally lead to symmetric weights (Kendall et al., 2020), neural networks with unidirectional weights are in general easier to implement in neuromorphic hardware.

In this setting, the dynamics of Equation (1) is changed into the more general form:

(9) $$\begin{array}{l} s_{t+1}=F\left(x,s_{t},\theta \right), \end{array}$$

and the conventionally proposed learning rule reads:

(10) $$\begin{array}{l} \;\Delta \theta =\eta {\hat{\nabla }}^{\text{VF}}\left(\beta \right),\text{ where}\\ \begin{array}{l} {\hat{\nabla }}^{\text{VF}}\left(\beta \right)\overset{\Delta }{=}\frac{1}{\beta }\frac{\partial F}{\partial \theta }{\left(x,s_{*},\theta \right)}^{⊤}\cdot \left(s_{*}^{\beta }-s_{*}\right), \end{array} \end{array}$$

where VF stands for Vector Field (Scellier et al., 2018). If the transition function *F* derives from a primitive function Φ (i.e., if $F=\frac{\partial \Phi }{\partial s}$), then ${\hat{\nabla }}^{\text{VF}}\left(\beta \right)$ is equal to ${\hat{\nabla }}^{\text{EP}}\left(\beta \right)$ in the limit β → 0 (i.e., ${\text{lim}}_{\beta \to 0}{\hat{\nabla }}^{\text{VF}}\left(\beta \right)={\text{lim}}_{\beta \to 0}{\hat{\nabla }}^{\text{EP}}\left(\beta \right)$).

## 3. Improving EP Training

We have seen in Equation (6) that the temporal variations of the network over the second phase of EP exactly compute BPTT gradients in the limit β → 0. This result appears to underpin the use of two phases as a fundamental element of EP, but is it really the case? In this section, we revisit EP as a gradient estimation procedure and propose an implementation in three phases instead of two. Moreover, we show how to optimize the cross-entropy loss function with EP. Combining these two new techniques enabled us to achieve the best performance on CIFAR-10 by EP, on architectures with bidirectional and unidirectional forward and backward connections (section 4).

### 3.1. Reducing Bias and Variance in the Gradient Estimate of the Loss Function

In the foundational work on EP, Scellier and Bengio (2017) demonstrate that:

(11) $$\begin{array}{l} \frac{d}{d\beta }{|}_{\beta =0}\frac{\partial \Phi }{\partial \theta }\left(x,s_{*}^{\beta },\theta \right)=-\frac{\partial L^{*}}{\partial \theta }. \end{array}$$

The traditional implementation of EP evaluates the left-hand side of Equation (11) using the estimate ${\hat{\nabla }}^{\text{EP}}\left(\beta \right)$ with two points β = 0 and β > 0, thereby calling for the need of two phases—the free phase and the nudged phase. However, the use of β > 0 in practice induces a systematic first order bias in the gradient estimation provided by EP. In order to eliminate this bias, we propose to perform a third phase with −β as the nudging factor, keeping the first and second phases unchanged. We then estimate the gradient of the loss using the following symmetric difference estimate:

(12) $$\begin{array}{l} {\hat{\nabla }}_{\text{sym}}^{\text{EP}}\left(\beta \right)\overset{\Delta }{=}\frac{1}{2\beta }\left(\frac{\partial \Phi }{\partial \theta }\left(x,s_{*}^{\beta },\theta \right)-\frac{\partial \Phi }{\partial \theta }\left(x,s_{*}^{-\beta },\theta \right)\right). \end{array}$$

Indeed, under mild assumptions on the function $\beta \mapsto \frac{\partial \Phi }{\partial \theta }\left(x,s_{*}^{\beta },\theta \right)$, we can show that, as β → 0:

(13) $$\begin{array}{l} \frac{{\hat{\nabla }}^{\text{EP}}\left(\beta \right)+{\hat{\nabla }}^{\text{EP}}\left(-\beta \right)}{2}=-\frac{\partial L^{*}}{\partial \theta }+O\left(\beta^{2}\right), \end{array}$$

(14) $$\begin{array}{l} {\hat{\nabla }}_{\text{sym}}^{\text{EP}}\left(\beta \right)=-\frac{\partial L^{*}}{\partial \theta }+O\left(\beta^{2}\right). \end{array}$$

This result is proved in Lemma 2 of the Supplementary Material (section 2). Equation (13) shows that the estimate ${\hat{\nabla }}^{\text{EP}}\left(\beta \right)$ possesses a first-order error term in β which the symmetric estimate ${\hat{\nabla }}_{\text{sym}}^{\text{EP}}\left(\beta \right)$ eliminates (Equation 14). Note that the first-order term of ${\hat{\nabla }}^{\text{EP}}\left(\beta \right)$ could also be canceled out on average by choosing the sign of β at random with even probability (so that 𝔼(β) = 0, see Algorithm 1 of the Supplementary Material, section 3.1). Although not explicitly stated in this purpose, the use of such randomization has been reported in some earlier publications on the MNIST task (Scellier and Bengio, 2017; Ernoult et al., 2020). However, in this work, we show that this method exhibits high variance in the training procedure.

**Figure 2:**
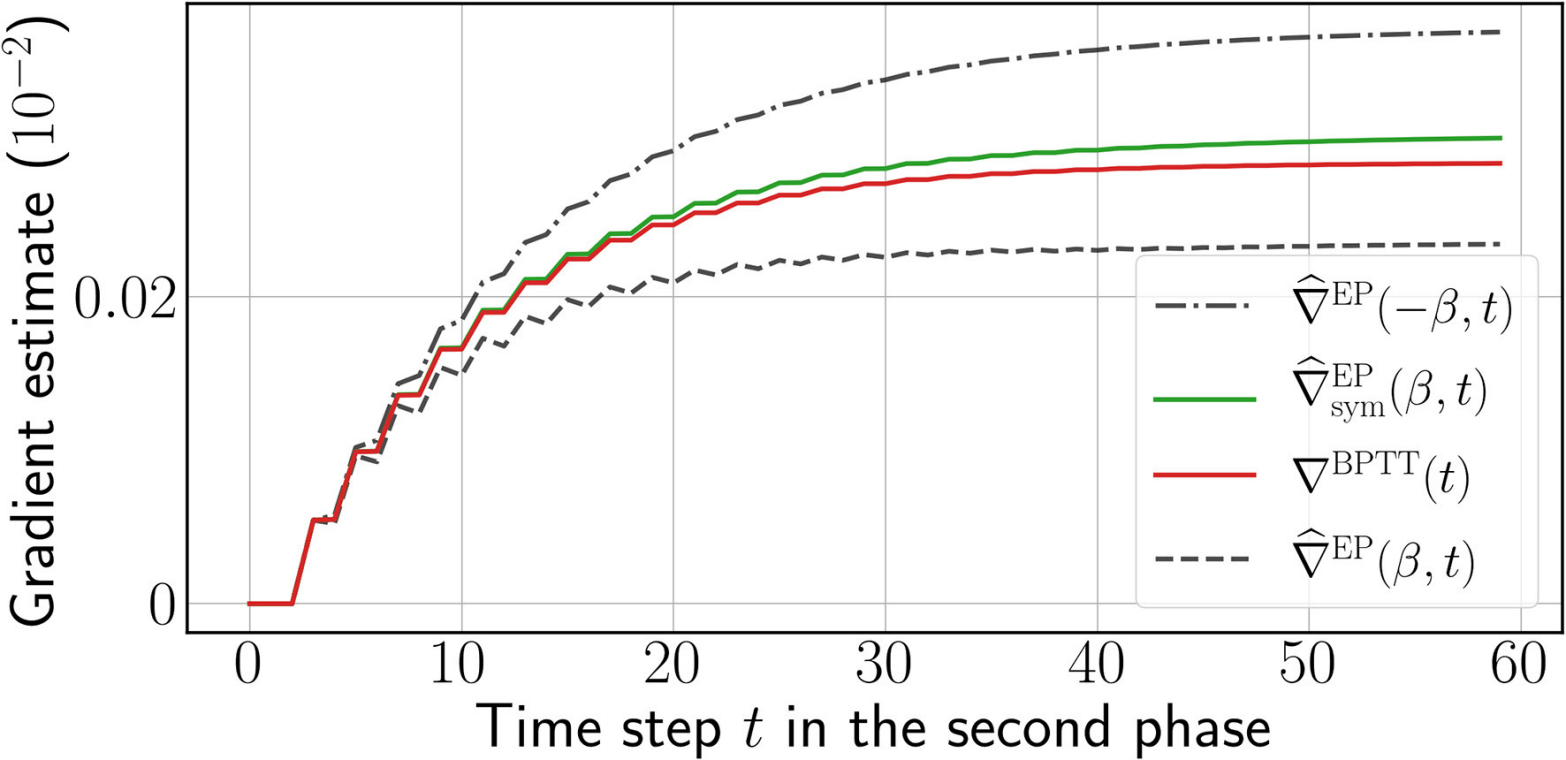
One-sided EP gradient estimate for opposite values of β = 0.1 (black dashed curves), symmetric EP gradient estimate (green curve), and reference gradients computed by BPTT (red curve) computed over the second phase, for a single weight chosen at random. The time step *t* is defined for BPTT and EP according to Equation (6). More instances can be found in Supplementary Material (section 6).

We call ${\hat{\nabla }}^{\text{EP}}\left(\beta \right)$ and ${\hat{\nabla }}_{\text{sym}}^{\text{EP}}\left(\beta \right)$ the one-sided and symmetric EP gradient estimates, respectively. The qualitative difference between these estimates is depicted in Figure 2, and the full training procedure is depicted in Algorithm 2 of the Supplementary Material (section 3.2).

Finally, this technique can also be applied to the Vector Field setting introduced in section 2.4 and we denote ${\hat{\nabla }}_{\text{sym}}^{\text{VF}}\left(\beta \right)$ the resulting symmetric estimate—see the Supplementary Material (section 4.3) for details.

### 3.2. Changing the Loss Function

We also introduce a novel architecture to optimize the cross-entropy loss with EP, narrowing the gap with conventional deep learning architectures for classification tasks. In the next paragraph, we denote $\hat{y}$ the set of neurons that carries out the prediction of the neural network.

#### 3.2.1. Squared Error Loss Function

**Figure 3:**
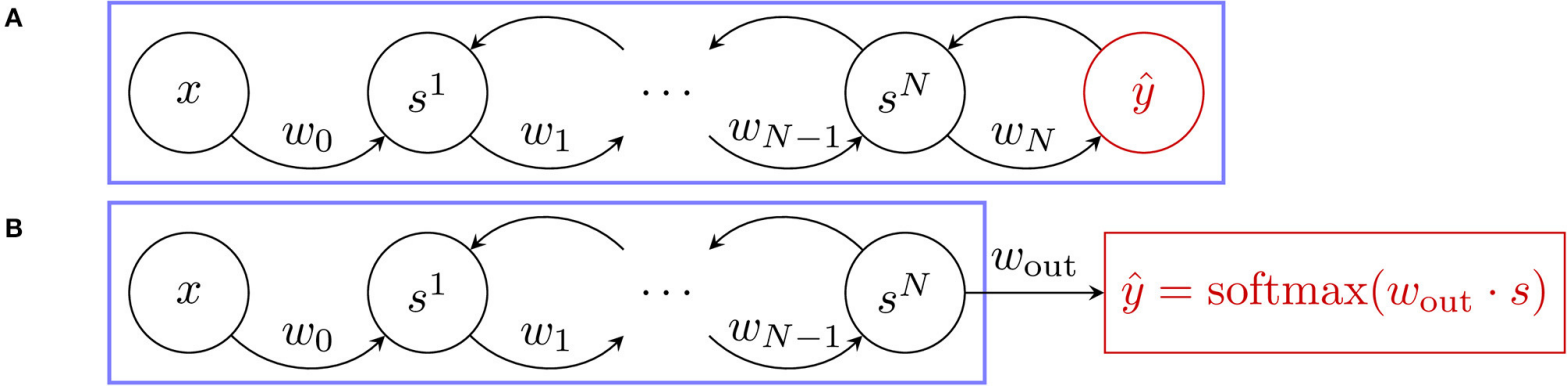
Free dynamics of the architectures used for the two loss functions where the blue frame delimits the system. **(A)** Squared Error loss function. The usual setting where the predictor ŷ (in red) takes part in the free dynamics of the neural network through bidirectional synaptic connections. **(B)** Cross-entropy loss function. The new approach proposed in this work where the predictor ŷ (also in red) is no longer involved in the system free dynamics and is implemented as a softmax readout.

Previous implementations of EP used the squared error loss. Using this loss function for EP is natural, as in this setting, the output $\hat{y}$ is viewed as a part of *s* (the state variable of the network), which can influence the state of the network through bidirectional synaptic connections (see Figure 3). Moreover, the nudging term in this case can be physically interpreted since it reads as an elastic force. The state of the network is of the form $s=\left(s^{1},\ldots ,s^{N},\hat{y}\right)$ where *h* = (*s*^1^, …, *s*^*N*^) represent the “hidden layers,” and the corresponding cost function is

(15) $$\begin{array}{l} \ell \left(\hat{y},y\right)=\frac{1}{2}\|\hat{y}-y{\|}^{2}. \end{array}$$

The second phase dynamics of the hidden state and output layer given by Equation (3) read, in this context:

(16) $$\begin{array}{l} {\;h}_{t+1}^{\beta }=\frac{\partial \Phi }{\partial h}\left(x,h_{t}^{\beta },{\hat{y}}_{t}^{\beta },\theta \right),\\ \begin{array}{l} {\hat{y}}_{t+1}^{\beta }=\frac{\partial \Phi }{\partial \hat{y}}\left(x,h_{t}^{\beta },{\hat{y}}_{t}^{\beta },\theta \right)+\beta \left(y-{\hat{y}}_{t}^{\beta }\right). \end{array} \end{array}$$

#### 3.2.2. Softmax Readout, Cross-Entropy Loss Function

In this paper, we propose an alternative approach, where the output $\hat{y}$ is not a part of the state variable *s* but is instead implemented as a read-out (see Figure 3), which is a function of *s* and of a weight matrix *w*_out_ of size dim(*y*) × dim(*s*). In practice, *w*_out_ reads out the last convolutional layer. At each time step *t* we define:

(17) $$\begin{array}{l} {\hat{y}}_{t}=\text{softmax}\left(w_{\text{out}}\cdot s_{t}\right). \end{array}$$

The cross-entropy cost function associated with the softmax readout is then:

(18) $$\begin{array}{l} \ell \left(s,y,w_{\text{out}}\right)=-\overset{C}{\underset{c=1}{\sum }}y_{c}\text{ log}\left({\text{softmax}}_{c}\left(w_{\text{out}}\cdot s\right)\right). \end{array}$$

Using $\frac{\partial \ell }{\partial s}\left(s,y,w_{\text{out}}\right)=w_{\text{out}}^{⊤}\cdot \left(\text{softmax}\left(w_{\text{out}}\cdot s\right)-y\right)$, the second phase dynamics given by Equation (3) read in this context:

(19) $$\begin{array}{l} s_{t+1}^{\beta }=\frac{\partial \Phi }{\partial s}\left(x,s_{t}^{\beta },\theta \right)+\beta w_{\text{out}}^{⊤}\cdot \left(y-{\hat{y}}_{t}^{\beta }\right). \end{array}$$

Note here that the loss $L^{*}=\ell \left(s_{*},y,w_{\text{out}}\right)$ also depends on the parameter *w*_out_. The Supplementary Material (section 4.2.2) provides the learning rule applied to *w*_out_.

### 3.3. Changing the Learning Rule of EP With Unidirectional Synaptic Connections

In the case of architectures with unidirectional connections, applying the traditional EP learning rule directly, as given by Equation (10), prescribes different forward and backward weights updates, resulting in significantly different forward and backward weights throughout learning. However, the theoretical equivalence between EP and BPTT only holds for bidirectional connections. Until now, training experiments of unidirectional weights EP have performed worse than bidirectional weights EP (Ernoult et al., 2020). In this work, therefore, we tailor a new learning rule for unidirectional weights, described in detail the Supplementary Material (section 4.3), where the forward and backward weights undergo the same weight updates, incorporating an equal leakage term. This way, forward and backward weights, although they are independently initialized, naturally converge to identical values throughout the learning process. A similar methodology, adapted from Kolen and Pollack (1994), has been shown to improve the performance of Feedback Alignment in Deep ConvNets (Akrout et al., 2019).

Assuming general dynamics of the form of Equation (9), we distinguish forward connections θ_f_ from backward connections θ_b_ so that θ = {θ_f_, θ_b_}, with θ_f_ and θ_b_ having same dimension. Assuming a first phase, a second phase with β > 0 and a third phase with −β, we define:

(20) $$\begin{array}{l} \forall \text{i}\in \left\{\text{f},\text{b}\right\},\\ \bar{\nabla_{\theta_{\text{i}}}^{\text{VF}}}\left(\beta \right)=\frac{1}{2\beta }\left({\frac{\partial F}{\partial \theta_{\text{i}}}}^{⊤}\left(x,s_{*}^{\beta },\theta \right)\cdot s_{*}^{\beta }-{\frac{\partial F}{\partial \theta_{\text{i}}}}^{⊤}\left(x,s_{*}^{-\beta },\theta \right)\cdot s_{*}^{-\beta }\right) \end{array}$$

and we propose the following update rules:

(21) $$\begin{array}{l} \;\{\begin{array}{c} \Delta \theta_{\text{f}}=\eta \left({\hat{\nabla }}_{\text{sym}}^{\text{KP-VF}}\left(\beta \right)-\lambda \theta_{\text{f}}\right)\\ \Delta \theta_{\text{b}}=\eta \left({\hat{\nabla }}_{\text{sym}}^{\text{KP-VF}}\left(\beta \right)-\lambda \theta_{\text{b}}\right) \end{array},\\ \begin{array}{l} \text{ with }{\hat{\nabla }}_{\text{sym}}^{\text{KP-VF}}\left(\beta \right)=\frac{1}{2}\left(\bar{\nabla_{\theta_{\text{f}}}^{\text{VF}}}\left(\beta \right)+\bar{\nabla_{\theta_{\text{b}}}^{\text{VF}}}\left(\beta \right)\right) \end{array} \end{array}$$

where η is the learning rate and λ a leakage parameter. The estimate ${\hat{\nabla }}_{\text{sym}}^{\text{KP-VF}}\left(\beta \right)$ can be thought of a generalization of Equation (12), as highlighted in the Supplementary Material (section 4.3) with an explicit application of Equation (21) to a ConvNet. In the case of a fully connected layer, both terms in the sum in the right hand side of Equation (21) are equal: ∂*F*/∂θ_i_ only depends on the neuron activations and not on θ_i_, in the same way, as seen at the end of section 2.3, that Equation (8) yields a fully local learning rule. The case of convolutional layers is a little more subtle, due to presence of the maximum pooling operations. The forward weights are involved in a pooling operation while the backward weights are involved in an unpooling operation. However, for the parameter update to be the same, the pooling and unpooling operations need to share information regarding the indices of maxima. Therefore, there is indeed a need for information transfer between backward and forward parameters, but this exchange is limited to the index of the maximum identified in the maximum pooling operation (this can be seen from Equation 24).

## 4. Results

In this section, we implement EP with the modifications described in section 3 and successfully train deep ConvNets on the CIFAR-10 vision task (Krizhevsky et al., 2009). The convolutional architecture used consists of four 3 × 3 convolutional layers of respective feature maps 128–256–512–512. We use a stride of one for each convolutional layer, and zero-padding of one for each layer except for the last layer. Each layer is followed by a 2 × 2 Max Pooling operation with a stride of two. The resulting flattened feature vector is of size 512. The weights are initialized using the default initialization of PyTorch, which is the uniform Kaiming initialization of He et al. (2015). The data is normalized and augmented with random horizontal flips and random crops. The training is performed with stochastic gradient descent with momentum and weight decay. We use the learning rate scheduler introduced by Loshchilov and Hutter (2016) to speed up convergence.

The hyper-parameters are reported in Table 1. All experiments are performed using PyTorch 1.4.0. (Paszke et al., 2017). The simulations were carried across several servers consisting of 14 Nvidia GeForce RTX 2080 TI GPUs in total. Each run was performed on a single GPU for an average run time of 2 days.

**Table 1:** Hyper-parameters used for the CIFAR-10 experiments.

**Hyper-parameter**	**Squared error**	**Cross-entropy**
*T*	250	250
*K*	30	25
β	0.5	1.0
Batch size	128	128
Initial learning rates (Layer-wise)	0.25 - 0.15 - 0.1 - 0.08 - 0.05	0.25 - 0.15 - 0.1 - 0.08 - 0.05
Final learning rates	10^−5^	10^−5^
Weight decay (All layers)	3·10^−4^	3·10^−4^
Momentum	0.9	0.9
Epoch	120	120
Cosine annealing Decay time (epochs)	100	100

### 4.1. ConvNets With Bidirectional Connections

We first consider the bidirectional weight setting of section 2.3. In Table 2, we compare the performance achieved by the ConvNet for each EP gradient estimate introduced in section 3.1 with the performance achieved by BPTT.

**Table 2:** Performance comparison on CIFAR-10 between BPTT and EP with several gradient estimation schemes.

**Loss function**	**EP gradient**	**EP error (%)**		**BPTT error (%)**	
	**estimate**	**Test**	**Train**	**Test**	**Train**
Squared error	2-Phase/${\hat{\nabla }}^{\text{EP}}$	86.64 (5.82)	84.90	11.10 (0.21)	3.69
	Random Sign	21.55 (20.00)	20.01		
	3-Phase/${\hat{\nabla }}_{\text{sym}}^{\text{EP}}$	12.45 (0.18)	7.83		
Cross-Ent.	3-Phase/${\hat{\nabla }}_{\text{sym}}^{\text{EP}}$	**11.68 (0.17)**	**4.98**	11.12 (0.21)	2.19
Cross-Ent. (Dropout)	3-Phase/${\hat{\nabla }}_{\text{sym}}^{\text{EP}}$	11.87 (0.29)	6.46	10.72 (0.06)	2.99
Cross-Ent.	3-Phase/${\hat{\nabla }}_{\text{sym}}^{\text{VF}}$	75.47 (4.72)	78.04	9.46 (0.17)	0.80
	3-Phase/${\hat{\nabla }}_{\text{sym}}^{\text{KP-VF}}$	**13.15 (0.49)**	8.87		

*Note that the different gradient estimates only apply to EP. We indicate over five trials the mean and standard deviation in parenthesis for the test error, and the mean train error*.

**Figure 4:**
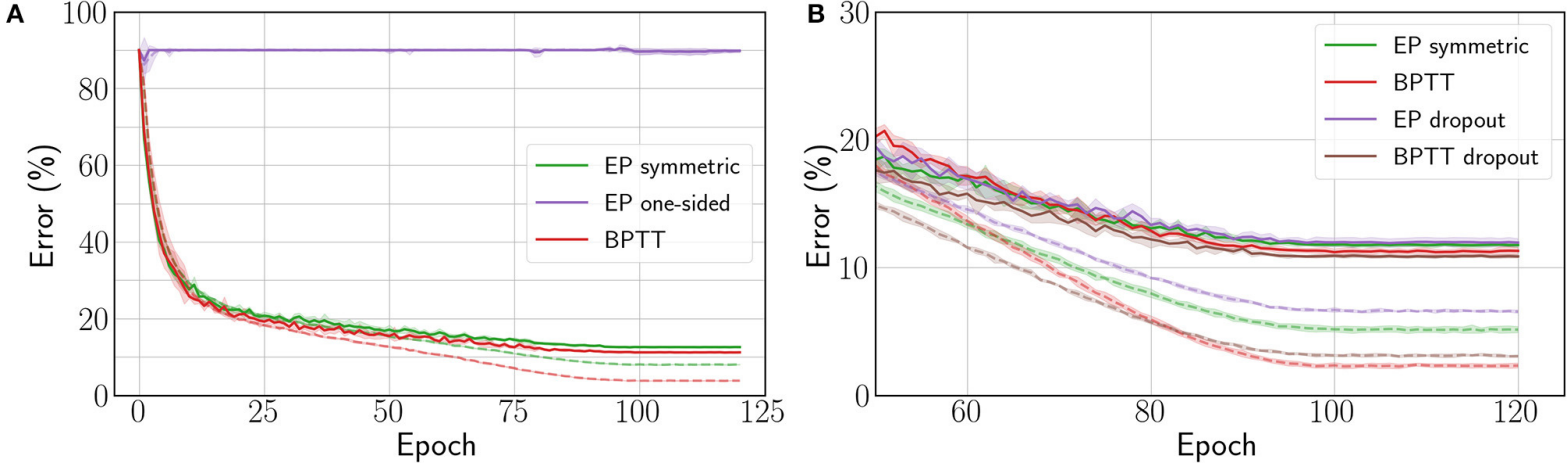
**(A)** Train (dashed) and test (solid) errors on CIFAR-10 with the Squared Error loss function. **(B)** Train (dashed) and test (solid) errors on CIFAR-10 with the Cross-Entropy loss function. The curves are averaged over 5 runs and shadows stand for ±1 × standard deviation. The change in error rate around epochs 85–90 is due to the end of the learning rate scheduler decay phase (Cosine annealing).

The one-sided gradient estimate leads to unstable training behavior where the network is unable to fit the data, as shown by the purple curve of Figure 4A, with 86.64% test error on CIFAR-10. When the bias in the gradient estimate is averaged out by choosing at random the sign of β during the second phase, the average test error over five runs goes down to 21.55% (see Table 2). However, one run among the five yielded instability similar to the one-sided estimate, whereas the four remaining runs lead to 12.61% test error and 8.64% train error. This method for estimating the loss gradient thus presents high variance—further experiments shown in the Supplementary Material (section 4.4) confirm this trend.

Conversely, the three-phase symmetric estimate enables EP to consistently reach 12.45% test error, with only 1.35% degradation with respect to BPTT (see Figure 4A). Therefore, removing the first-order error term in the gradient estimate is critical for scaling to deeper architectures. Proceeding to this end deterministically (with three phases) rather than stochastically (with a randomized nudging sign) appears more reliable.

The results of Table 2 also show that the readout scheme introduced in section 3.2 to optimize the cross-entropy loss function enables EP to narrow the performance gap with BPTT down to 0.56% while outperforming the Squared Error setting by 0.77%. However, we observe that the test errors reached by BPTT are similar for the squared error and the cross-entropy loss. The fact that only EP benefits from the cross-entropy loss is due to the output not being part of the dynamics, which reduces the number of layers following the dynamics by one.

We also adapted dropout (Srivastava et al., 2014) to convergent RNNs (see the Supplementary Material, section 4.5 for implementation details) to see if the performance could be improved further. However, we can observe from Table 2 and Figure 4B that contrary to BPTT, the EP test error is not improved by adding a 0.1 dropout probability in the neuron layer after the convolutions.

### 4.2. ConvNets With Unidirectional Connections

We now present the accuracy achieved by EP when the architecture uses distinct forward and backward weights, using a softmax readout. For this architecture, the backward weights are defined for all convolutional layers, except the first convolutional layer connected to the static input. The forward and backward weights are initialized randomly and independently at the beginning of training. The backward weights have no bias contrary to their forward counterparts. The hyper-parameters such as learning rate, weight decay and momentum are shared between forward and backward weights.

**Figure 5:**
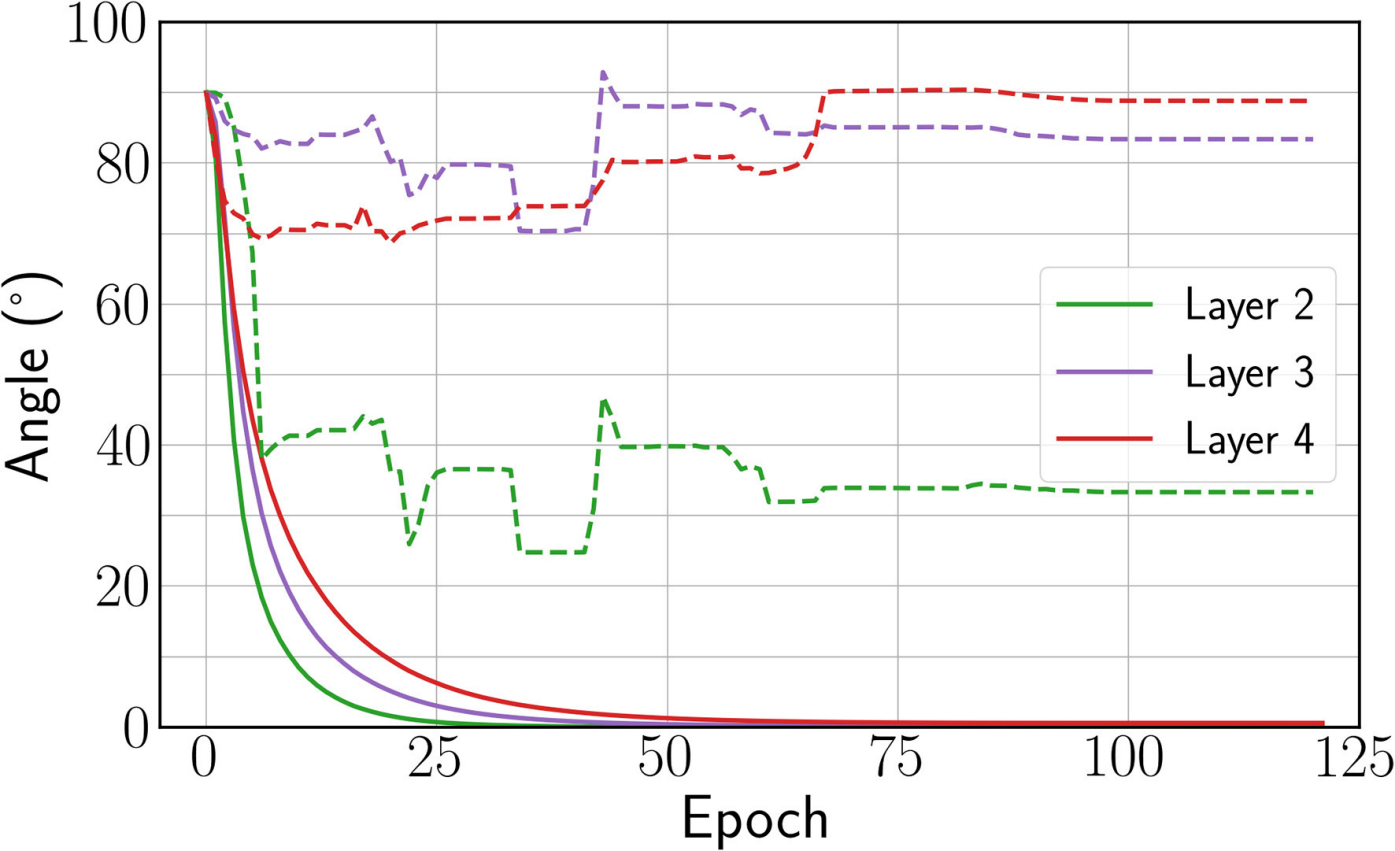
Angle between forward and backward weights for the new estimate ${\hat{\nabla }}_{\text{sym}}^{\text{KP-VF}}$ introduced (solid) and ${\hat{\nabla }}_{\text{sym}}^{\text{VF}}$ (dashed). The angle is not defined for the first layer because the input layer is clamped.

As seen in Table 2, we find that the estimate ${\hat{\nabla }}_{\text{sym}}^{\text{VF}}\left(\beta \right)$ leads to a poor performance with 75.47% test-error. We concomitantly observed that forward and backward weight did not align well, as shown by the dashed curves in Figure 5. Conversely, when using our new estimate ${\hat{\nabla }}_{\text{sym}}^{\text{KP-VF}}\left(\beta \right)$ defined in section 3.3, a good performance is recovered with only 1.5% performance degradation with respect to the architecture with bidirectional connections, and a 3% degradation with respect to BPTT (see Table 2). The discrepancy between the BPTT test error achieved by the architecture with bidirectional (11.12%) and unidirectional (9.46%) connections comes from the increase in parameters provided by backward weights. As observed in the weight alignment curves in Figure 5, forward and backward weights are well-aligned by epoch 50 when using the new estimate. These results suggest that enhancing forward and backward weights alignment can help EP training in deep ConvNets.

## 5. Discussion

Our results unveil the necessity, in order to scale EP to deep convolutional neural networks on hard visual tasks, to compute better gradient estimates than the conventional implementation of EP. This traditional implementation incorporates a first order gradient estimate bias, which severely impedes the training of deep architectures. Conversely, we saw that the three-phase EP proposed here removes this bias and brings EP performance on CIFAR-10 close to the one achieved by BPTT. Additionally, our new technique to train EP with softmax readout reduces the gap between EP and BPTT further down to 0.56%, while maintaining the locality of the learning rule of all parameters.

While the test accuracy of BPTT and our adapted EP are very close, we can notice in Table 2 that BPTT fits the training data better than EP by at least 2.8%. Also, the introduction of dropout improves BPTT performance, while it has no significant effect on the test accuracy of EP. These two insights combined suggest that EP training may have a self-regularizing effect applied throughout the network, similar to the effects of dropout. We hypothesize this effect to be not only due to the residual estimation bias of the BPTT gradients by EP, but also to an additional inherent error term due to the fact that in practice, the fixed point is approached with a precision that depends on the number of time steps at inference. While the exactness of the fixed point is crucial for EP, BPTT computes exact gradients regardless of whether the fixed point is not exactly reached.

We also saw that employing a new training technique that still preserves the spatial locality of EP computations—and therefore its suitability for neuromorphic implementations—our results extend to the case of an architecture with distinct forward and backward synaptic connections. We only observe a 1.5% performance degradation with respect to the bidirectional architecture. This result demonstrates the scalability of EP without the biologically implausible requirement of a bidirectional connectivity pattern.

Our three steady states-based gradient estimate comes at a computational cost with regards to the conventional EP implementation, as an additional phase is needed. Even though the steady state of the free phase s_*_ is not used to compute the gradient estimate in Equation (12), we experimentally found that s_*_ is needed as a starting point for the second and third phases. In terms of simulation time, EP is 20% slower than BPTT due to the dynamics performed in second and third phases. However, the memory requirement to store the computational graph unfolded in time in the case of BPTT far outweighs the memory needed by EP, which consists only of the steady states reached by the neurons.

The full potential of EP will be best envisioned on neuromorphic hardware. Multiple works have investigated the implementation of EP on such systems (Ernoult et al., 2019, 2020; Foroushani et al., 2020; Ji and Gross, 2020; Zoppo et al., 2020), in both rate based (Kendall et al., 2020) and spiking approaches (Martin et al., 2020). Most of these approaches employ analog circuits that exploit device physics to implement the dynamics of EP intrinsically. The spatially local nature of EP computations, on top of its connection with physical equations, make this mapping between EP and neuromorphic hardware natural. Our prescription to run two nudging phases with opposite nudging strengths could be implemented naturally in neuromorphic systems. In fact, the use of differential operation to cancel inherent biases is a technique widely used in electronics, and in neuromorphic computing in particular (Hirtzlin et al., 2019). Overall, our work provides evidence that EP is a compelling approach to scale neuromorphic on-chip training to real-world tasks in a fully local fashion.

## Data Availability Statement

The original contributions presented in the study are publicly available. This data can be found here: https://github.com/Laborieux-Axel/Equilibrium-Propagation.

## Author Contributions

AL developed the PyTorch code for the project and performed the simulations. ME supervised the work, helped debug the code, guided hyperparameter search, and designed the experiments with unidirectional connections. BS proposed the ideas of unbiasing the gradient estimate and of using a softmax readout. DQ, JG, and YB provided additional guidance and support. All authors participated in data analysis, discussed the results, and co-edited the manuscript.

## Conflict of Interest

The authors declare that the research was conducted in the absence of any commercial or financial relationships that could be construed as a potential conflict of interest.

## Footnotes

^1^In the original version of EP for real-time dynamical systems (Scellier and Bengio, 2017), the dynamics derive from an energy function *E*, which plays a similar role to the primitive function Φ in the discrete-time setting studied here.
