# Supplementary material for: Scaling Equilibrium Propagation to Deep ConvNets by Drastically Reducing Its Gradient Estimator Bias
Source: Front Neurosci. 2021 Feb 18;15:633674. doi: 10.3389/fnins.2021.633674 (PMC7930909; doi:10.3389/fnins.2021.633674)
Supplement: Supplementary file 1 [file Data_Sheet_1.pdf]

frontiers

Supplementary Material

1 GRADIENTS OF BPTT

In this appendix, we define $\nabla^{\mathrm{BPTT}}(t)$, the gradient computed by BPTT truncated to the last t time steps $(T-t,\ldots,T)$. To do this, let us rewrite Eq. (1) as $s_{t+1} = \frac{\partial \Phi}{\partial s}(x, s_t, \theta_t = \theta)$, where θ_t denotes the parameter at time step t, the value θ being shared across all time steps. We consider the loss after T time steps $\mathcal{L} = \ell(s_T, y)$. Rewriting the dynamics in such a way enables us to define $\frac{\partial \mathcal{L}}{\partial \theta_t}$ as the sensitivity of the loss with respect to θ_t, when $\theta_0,\ldots,\theta_{t-1},\theta_{t+1},\ldots,\theta_{T-1}$ remain fixed (set to the value θ). With these notations, the gradient computed by BPTT truncated to the last t time steps is

$$\nabla^{\mathrm{BPTT}}(t) = \frac{\partial \mathcal{L}}{\partial \theta_{T-t}} + \ldots + \frac{\partial \mathcal{L}}{\partial \theta_{T-1}}. \tag{S1}$$

2 ERROR TERMS IN THE ESTIMATES OF THE LOSS GRADIENT

In this appendix, we prove Lemma 2 which shows that $\widehat{\nabla}^{\mathrm{EP}}_{\mathrm{sym}}(\beta)$ is a better estimate of $-\frac{\partial \mathcal{L}^*}{\partial \theta}$ than $\widehat{\nabla}^{\mathrm{EP}}(\beta)$. First, we recall the theorem proved in Scellier and Bengio (2017).

THEOREM 1 (Scellier and Bengio (2017)).

$$\left.\frac{d}{d\beta}\right|_{\beta=0} \frac{\partial \Phi}{\partial \theta}(x, s_*^\beta, \theta) = -\frac{\partial \mathcal{L}^*}{\partial \theta}. \tag{S2}$$

We also recall that the two estimates (one-sided and symmetric) are, by definition:

$$\widehat{\nabla}^{\mathrm{EP}}(\beta) \triangleq \frac{1}{\beta}\left(\frac{\partial \Phi}{\partial \theta}(x, s_*^\beta, \theta) - \frac{\partial \Phi}{\partial \theta}(x, s_*, \theta)\right),$$
$$\widehat{\nabla}^{\mathrm{EP}}_{\mathrm{sym}}(\beta) \triangleq \frac{1}{2\beta}\left(\frac{\partial \Phi}{\partial \theta}(x, s_*^\beta, \theta) - \frac{\partial \Phi}{\partial \theta}(x, s_*^{-\beta}, \theta)\right).$$

Finally we recall Lemma 2, for readability.

LEMMA 2. *Provided the function $\beta \mapsto \frac{\partial \Phi}{\partial \theta}(x, s_*^\beta, \theta)$ is three times differentiable, we have, as $\beta \to 0$:*

$$\widehat{\nabla}^{\mathrm{EP}}(\beta) = -\frac{\partial \mathcal{L}^*}{\partial \theta} + \frac{\beta}{2}\left.\frac{d^2}{d\beta^2}\right|_{\beta=0} \frac{\partial \Phi}{\partial \theta}(s_*^\beta, \theta) + O(\beta^2),$$
$$\widehat{\nabla}^{\mathrm{EP}}_{\mathrm{sym}}(\beta) = -\frac{\partial \mathcal{L}^*}{\partial \theta} + O(\beta^2).$$

PROOF OF LEMMA 2. Let us define

$$f(\beta) \triangleq \frac{\partial \Phi}{\partial \theta}(x, s_*^\beta, \theta).$$

The formula of Theorem 1 rewrites

$$f'(0) = -\frac{\partial \mathcal{L}^*}{\partial \theta}.$$

As $\beta \to 0$, we have the Taylor expansion

$$f(\beta) = f(0) + \beta f'(0) + \frac{\beta^2}{2} f''(0) + O(\beta^3). \tag{S3}$$

With these notations, the one-sided estimate reads

$$\begin{aligned}\widehat{\nabla}^{\mathrm{EP}}(\beta) &= \frac{1}{\beta}\left(f(\beta) - f(0)\right) \\ &= f'(0) + \frac{\beta}{2} f''(0) + O(\beta^2) \\ &= -\frac{\partial \mathcal{L}^*}{\partial \theta} + \frac{\beta}{2} \left.\frac{d^2}{d\beta^2}\right|_{\beta=0} \frac{\partial \Phi}{\partial \theta}(x, s_*^\beta, \theta) + O(\beta^2).\end{aligned}$$

We can also write a Taylor expansion around 0 at the point $-\beta$. We have

$$f(-\beta) = f(0) - \beta f'(0) + \frac{\beta^2}{2} f''(0) + O(\beta^3). \tag{S4}$$

Subtracting Eq. S4 from Eq. S3, we can rewrite the symmetric difference estimate as

$$\begin{aligned}\widehat{\nabla}^{\mathrm{EP}}_{\mathrm{sym}}(\beta) &= \frac{1}{2\beta}\left(f(\beta) - f(-\beta)\right) \\ &= f'(0) + O(\beta^2) \\ &= -\frac{\partial \mathcal{L}^*}{\partial \theta} + O(\beta^2).\end{aligned}$$

The derivative to the third order of f is only used to get the $O(\beta^3)$ term in the expansion Eq. (S3), it can be changed into $o(\beta^2)$ if we only assume f twice differentiable.

3 PSEUDO CODE

3.1 Random one-sided estimation of the loss gradient

In this appendix, we define the random one-sided estimation used in this work and by Scellier and Bengio (2017); Ernoult et al. (2020).

Algorithm 1 EP with random one-sided estimation of the loss gradient. We omit the activation function σ for clarity.

Input: x, y, θ, η.
Output: θ.

1: $s_0 \leftarrow 0$
2: **for** $t = 0$ to T **do** ▷ First phase.
3: $\quad s_{t+1} \leftarrow \frac{\partial \Phi}{\partial s}(x, s_t, \theta)$
4: **end for**
5: $s_* \leftarrow s_T$
6: $\beta \leftarrow \beta \times \mathrm{Bernoulli}(1, -1)$ ▷ Random sign.
7: $s_0^\beta \leftarrow s_*$
8: **for** $t = 0$ to K **do** ▷ Second phase.
9: $\quad s_{t+1}^\beta \leftarrow \frac{\partial \Phi}{\partial s}(x, s_t^\beta, \theta) - \beta \frac{\partial \ell}{\partial s}(s_t^\beta, y)$
10: **end for**
11: $s_*^\beta \leftarrow s_K^\beta$
12: $\nabla_\theta^{\mathrm{EP}} \leftarrow \frac{1}{\beta}\left(\frac{\partial \Phi}{\partial \theta}(s_*^\beta, \theta) - \frac{\partial \Phi}{\partial \theta}(s_*, \theta)\right)$
13: $\theta \leftarrow \theta + \eta \nabla_\theta^{\mathrm{EP}}$
14: **return** θ

3.2 Symmetric difference estimation of the loss gradient

In this appendix, we define the estimation procedure using a symmetric difference estimate introduced in this work.

Algorithm 2 EP with symmetric difference estimation of the loss gradient. We omit the activation function σ for clarity.

Input: x, y, θ, η.
Output: θ.

$s_0 \leftarrow 0$
for $t = 0$ to T **do**
  $s_{t+1} \leftarrow \frac{\partial \Phi}{\partial s}(x, s_t, \theta)$ ▷ First phase.
end for
$s_* \leftarrow s_T$ ▷ Store the free steady state.
$s_0^\beta \leftarrow s_*$
for $t = 0$ to K **do**
  $s_{t+1}^\beta \leftarrow \frac{\partial \Phi}{\partial s}(x, s_t^\beta, \theta) - \beta \frac{\partial \ell}{\partial s}(s_t^\beta, y)$ ▷ Second phase.
end for
$s_*^\beta \leftarrow s_K^\beta$
$s_0^{-\beta} \leftarrow s_*$ ▷ Back to the free steady state.
for $t = 0$ to K **do**
  $s_{t+1}^{-\beta} \leftarrow \frac{\partial \Phi}{\partial s}(x, s_t^{-\beta}, \theta) + \beta \frac{\partial \ell}{\partial s}(s_t^{-\beta}, y)$ ▷ Third phase.
end for
$s_*^{-\beta} \leftarrow s_K^{-\beta}$
$\widehat{\nabla}_\theta^{\mathrm{EP}} \leftarrow \frac{1}{2\beta}\left(\frac{\partial \Phi}{\partial \theta}(s_*^\beta, \theta) - \frac{\partial \Phi}{\partial \theta}(s_*^{-\beta}, \theta)\right)$
$\theta \leftarrow \theta + \eta \widehat{\nabla}_\theta^{\mathrm{EP}}$
return θ

4 CONVOLUTIONAL RECURRENT NEURAL NETWORKS

Throughout this section, N^{conv} and N^{fc} denote respectively the number of convolutional layers and fully connected layers in the convolutional RNN, and $N^{\mathrm{tot}} \triangleq N^{\mathrm{conv}} + N^{\mathrm{fc}}$. The neuron layers are denoted by s and range from $s^0 = x$ the input to the output $s^{N^{\mathrm{tot}}}$ in the case of squared error, or $s^{N^{\mathrm{tot}}-1}$ in the case of softmax read-out.

4.1 Definition of the operations

In this subsection we detail the operations involved in the dynamics of a convolutional RNN.

- The 2-D convolution between w with dimension $(C_{\mathrm{out}}, C_{\mathrm{in}}, F, F)$ and an input x of dimensions $(C_{\mathrm{in}}, H_{\mathrm{in}}, W_{\mathrm{in}})$ and stride one is a tensor y of size $(C_{\mathrm{out}}, H_{\mathrm{out}}, W_{\mathrm{out}})$ defined by:

$$y_{c,h,w} = (w \star x)_{c,h,w} = B_c + \sum_{i=0}^{C_{\mathrm{in}}-1} \sum_{j=0}^{F-1} \sum_{k=0}^{F-1} w_{c,i,j,k} x_{i,j+h,k+w}, \tag{S5}$$

  where B_c is a channel-wise bias.

- The 2-D transpose convolution of y by $\tilde{w}$ is then defined in this work as the gradient of the 2-D convolution with respect to its input:

$$(\tilde{w} \star y) \triangleq \frac{\partial (w \star x)}{\partial x} \cdot y \tag{S6}$$

- The dot product "•" generalized to pairs of tensors of same shape (C, H, W):

$$a \bullet b = \sum_{c=0}^{C-1} \sum_{h=0}^{H-1} \sum_{w=0}^{W-1} a_{c,h,w} b_{c,h,w}. \tag{S7}$$

- The pooling operation $\mathcal{P}$ with stride F and filter size F of x:

$$\mathcal{P}_F(x)_{c,h,w} = \max_{i,j \in [0, F-1]} \left\{ x_{c, F(h-1)+1+i, F(w-1)+1+j} \right\}, \tag{S8}$$

with relative indices of maximums within each pooling zone given by:

$$\mathrm{ind}_{\mathcal{P}}(x)_{c,h,w} = \underset{i,j \in [0, F-1]}{\mathrm{argmax}} \left\{ x_{c, F(h-1)+1+i, F(w-1)+1+j} \right\} = (i^*(x,h), j^*(x,w)). \tag{S9}$$

- The unpooling operation $\mathcal{P}^{-1}$ of y with indices $\mathrm{ind}_{\mathcal{P}}(x)$ is then defined as:

$$\mathcal{P}^{-1}(y, \mathrm{ind}_{\mathcal{P}}(x))_{c,h,w} = \sum_{i,j} y_{c,i,j} \cdot \delta_{h, F(i-1)+1+i^*(x,h)} \cdot \delta_{w, F(j-1)+1+j^*(x,w)}, \tag{S10}$$

which consists in filling a tensor with the same dimensions as x with the values of y at the indices $\mathrm{ind}_{\mathcal{P}}(x)$, and zeroes elsewhere. For notational convenience, we omit to write explicitly the dependence on the indices except when appropriate.

- The flattening operation $\mathcal{F}$ is defined as reshaping a tensor of dimensions (C, H, W) to $(1, CHW)$. We denote by $\mathcal{F}^{-1}$ its inverse.

4.2 Convolutional RNNs with symmetric connections

In this section, we write explicitly the dynamics and the learning rules applied for the convolutional architecture with symmetric connections, for the Squared loss function and the Cross-Entropy loss function, for the one-sided and symmetric estimates.

4.2.1 Squared Error loss

4.2.1.1 Equations of the dynamics.

In this case, the dynamics read:

$$\begin{cases} s_{t+1}^{n+1} = \sigma\left(\mathcal{P}(w_{n+1} \star s_t^n) + \tilde{w}_{n+2} \star \mathcal{P}^{-1}(s_t^{n+2})\right), \qquad \forall n \in [0, N^{\mathrm{conv}} - 2] \\ s_{t+1}^{N^{\mathrm{conv}}} = \sigma\left(\mathcal{P}(w_{N^{\mathrm{conv}}} \star s_t^{N^{\mathrm{conv}}-1}) + \mathcal{F}^{-1}({w_{N^{\mathrm{conv}}+1}}^\top \cdot s_t^{N^{\mathrm{conv}}+1})\right), \\ s_{t+1}^{N^{\mathrm{conv}}+1} = \sigma\left(w_{N^{\mathrm{conv}}+1} \cdot \mathcal{F}(s_t^{N^{\mathrm{conv}}}) + {w_{N^{\mathrm{conv}}+2}}^\top \cdot s_t^{N^{\mathrm{conv}}+2}\right), \\ s_{t+1}^{n+1} = \sigma\left(w_{n+1} \cdot s_t^n + {w_{n+2}}^\top \cdot s_t^{n+2}\right), \qquad \forall n \in [N^{\mathrm{conv}} + 1, N^{\mathrm{tot}} - 2] \\ s_{t+1}^{N^{\mathrm{tot}}} = \sigma\left(w_{N^{\mathrm{tot}}} \cdot s_t^{N^{\mathrm{tot}}-1}\right) + \beta(y - s^{N^{\mathrm{tot}}}), \quad \text{with } \beta = 0 \text{ during the first phase,} \end{cases} \tag{S11}$$

where we take the convention $s^0 = x$. In this case, we have $\hat{y} = s_{t+1}^{N^{\mathrm{tot}}}$. Considering the function:

$$\Phi(x, s^1, \cdots, s^{N^{\text{tot}}}) = \sum_{n=N_{\text{conv}}+2}^{N_{\text{tot}}-1} {s^{n+1}}^\top \cdot w_{n+1} \cdot s^n + {s^{N_{\text{conv}}+1}}^\top \cdot w_{N_{\text{conv}}+1} \cdot \mathcal{F}(s_t^{N_{\text{conv}}})$$
$$+ \sum_{n=1}^{N_{\text{conv}}-1} s^{n+1} \bullet \mathcal{P}\left(w_{n+1} \star s^n\right) + s^1 \bullet \mathcal{P}\left(w_1 \star x\right),$$

when ignoring the activation function, we have:

$$\forall n \in [1, N^{\text{tot}}]: \quad s_t^n \approx \frac{\partial \Phi}{\partial s^n}. \tag{S12}$$

Note that in the case of the Squared Error loss function, the dynamics of the output layer derive from Φ as it can be seen by Eq. (S12).

4.2.1.2 Learning rules for the one-sided EP estimator.

In this case, the learning rules read:

$$\begin{cases} \forall n \in [N_{\text{conv}}+2, N_{\text{tot}}-1]: \quad \Delta w_n = \frac{1}{\beta}\left(s_*^{n+1,\beta} \cdot {s_*^{n,\beta}}^\top - s_*^{n+1} \cdot {s_*^n}^\top\right) \\ \Delta w_{N_{\text{conv}}+1} = \frac{1}{\beta}\left(s_*^{N_{\text{conv}}+1,\beta} \cdot \mathcal{F}\left(s_*^{N_{\text{conv}},\beta}\right)^\top - s_*^{N_{\text{conv}}+1} \cdot \mathcal{F}\left(s_*^{N_{\text{conv}}}\right)^\top\right) \\ \forall n \in [1, N_{\text{conv}}-1]: \quad \Delta w_{n+1} = \frac{1}{\beta}\left(\mathcal{P}^{-1}(s_*^{n+1,\beta}) \star s_*^{n,\beta} - \mathcal{P}^{-1}(s_*^{n+1}) \star s_*^n\right) \\ \Delta w_1 = \frac{1}{\beta}\left(\mathcal{P}^{-1}(s_*^{1,\beta}) \star x - \mathcal{P}^{-1}(s_*^1) \star x\right) \end{cases}, \tag{S13}$$

4.2.1.3 Learning rules for the symmmetric EP estimator.

In this case, the learning rules read:

$$\begin{cases} \forall n \in [N_{\text{conv}}+2, N_{\text{tot}}-1]: \quad \Delta w_n = \frac{1}{2\beta}\left(s_*^{n+1,\beta} \cdot {s_*^{n,\beta}}^\top - s_*^{n+1,-\beta} \cdot {s_*^{n,-\beta}}^\top\right) \\ \Delta w_{N_{\text{conv}}+1} = \frac{1}{2\beta}\left(s_*^{N_{\text{conv}}+1,\beta} \cdot \mathcal{F}\left(s_*^{N_{\text{conv}},\beta}\right)^\top - s_*^{N_{\text{conv}}+1,-\beta} \cdot \mathcal{F}\left(s_*^{N_{\text{conv}},-\beta}\right)^\top\right) \\ \forall n \in [1, N_{\text{conv}}-1]: \quad \Delta w_{n+1} = \frac{1}{2\beta}\left(\mathcal{P}^{-1}(s_*^{n+1,\beta}) \star s_*^{n,\beta} - \mathcal{P}^{-1}(s_*^{n+1,-\beta}) \star s_*^{n,-\beta}\right) \\ \Delta w_1 = \frac{1}{2\beta}\left(\mathcal{P}^{-1}(s_*^{1,\beta}) \star x - \mathcal{P}^{-1}(s_*^{1,-\beta}) \star x\right) \end{cases}, \tag{S14}$$

4.2.2 Cross-Entropy loss

4.2.2.1 Equations of the dynamics.

In this case, the dynamics read:

$$
\begin{cases}
s_{t+1}^{n+1} = \sigma\left(\mathcal{P}(w_{n+1} \star s_t^{n}) + \tilde{w}_{n+2} \star \mathcal{P}^{-1}(s_t^{n+2})\right), \qquad \forall n \in [0, N^{\text{conv}} - 2] \\
s_{t+1}^{N^{\text{conv}}} = \sigma\left(\mathcal{P}(w_{N^{\text{conv}}} \star s_t^{N^{\text{conv}}-1}) + \mathcal{F}^{-1}({w_{N^{\text{conv}}+1}}^{\top} \cdot s_t^{N^{\text{conv}}+1})\right), \\
s_{t+1}^{N^{\text{conv}}+1} = \sigma\left(w_{N^{\text{conv}}+1} \cdot \mathcal{F}(s_t^{N^{\text{conv}}}) + {w_{N^{\text{conv}}+2}}^{\top} \cdot s_t^{N^{\text{conv}}+2}\right), \\
s_{t+1}^{n+1} = \sigma\left(w_{n+1} \cdot s_t^{n} + {w_{n+2}}^{\top} \cdot s_t^{n+2}\right), \qquad \forall n \in [N^{\text{conv}} + 1, N^{\text{tot}} - 3] \\
s_{t+1}^{N^{\text{tot}}-1} = \sigma\left(w_{N^{\text{tot}}-1} \cdot s_t^{N^{\text{tot}}-2}\right) + \beta {w_{\text{out}}}^{\top} \cdot (y - \hat{y}) \quad \text{with } \beta = 0 \text{ during the first phase,} \\
\hat{y} = \text{softmax}(w_{\text{out}} \cdot s_t^{N^{\text{tot}}-1}),
\end{cases}
\tag{S15}
$$

where we keep again the convention $s^0 = x$. Considering the function:

$$
\Phi(x, s^1, \cdots, s^{N^{\text{tot}}-1}) = \sum_{n=N_{\text{conv}}+1}^{N_{\text{tot}}-2} {s^{n+1}}^{\top} \cdot w_n \cdot s^n + s^{N_{\text{conv}}+1} \cdot w_{N_{\text{conv}}+1} \cdot \mathcal{F}(s_t^{N_{\text{conv}}})
$$
$$
+ \sum_{n=1}^{N_{\text{conv}}-1} s^{n+1} \bullet \mathcal{P}\left(w_{n+1} \star s^n\right) + s^1 \bullet \mathcal{P}\left(w_1 \star x\right),
$$

when ignoring the activation function, we have:

$$
\forall n \in [1, N^{\text{tot}} - 1]: \quad s_t^n \approx \frac{\partial \Phi}{\partial s^n}, \qquad \hat{y} = \text{softmax}(w_{\text{out}} \cdot s_t^{N^{\text{tot}}-1}). \tag{S16}
$$

Note that in this case and contrary to the Squared Error loss function, the dynamics of the output layer do not derive from the primitive function Φ, as it can be seen from Eq. (S16)

4.2.2.2 Learning rules for the one-sided EP estimator.

In this case, the learning rules read:

$$
\begin{cases}
\Delta w_{\text{out}} = -\left(\widehat{y}_*^{\beta} - y\right) \cdot {s_*^{\beta, N}}^{\top}. \\
\forall n \in [N_{\text{conv}} + 2, N_{\text{tot}} - 2]: \quad \Delta w_n = \frac{1}{\beta}\left(s_*^{n+1,\beta} \cdot {s_*^{n,\beta}}^{\top} - s_*^{n+1} \cdot {s_*^{n}}^{\top}\right) \\
\Delta w_{N_{\text{conv}}+1} = \frac{1}{\beta}\left(s_*^{N_{\text{conv}}+1,\beta} \cdot \mathcal{F}\left(s_*^{N_{\text{conv}},\beta}\right)^{\top} - s_*^{N_{\text{conv}}+1} \cdot \mathcal{F}\left(s_*^{N_{\text{conv}}}\right)^{\top}\right) \\
\forall n \in [1, N_{\text{conv}} - 1]: \quad \Delta w_{n+1} = \frac{1}{\beta}\left(\mathcal{P}^{-1}(s_*^{n+1,\beta}) \bullet s_*^{n,\beta} - \mathcal{P}^{-1}(s_*^{n+1}) \bullet s_*^{n}\right) \\
\Delta w_1 = \frac{1}{\beta}\left(\mathcal{P}^{-1}(s_*^{1,\beta}) \bullet x - \mathcal{P}^{-1}(s_*^{1}) \bullet x\right)
\end{cases}
. \tag{S17}
$$

4.2.2.3 Learning rules for the symmetric EP estimator.

In this case, the learning rules read:

$$\begin{cases} \Delta w_{\text{out}} = -\frac{1}{2}\left(\left(\widehat{y}_*^{\beta} - y\right) \cdot s_*^{\beta,N^\top} + \left(\widehat{y}_*^{-\beta} - y\right) \cdot s_*^{-\beta,N^\top}\right). \\ \forall n \in [N_{\text{conv}}+2, N_{\text{tot}}-2]: \quad \Delta w_n = \frac{1}{2\beta}\left(s_*^{n+1,\beta} \cdot s_*^{n,\beta^\top} - s_*^{n+1,-\beta} \cdot s_*^{n,-\beta^\top}\right) \\ \Delta w_{N_{\text{conv}}+1} = \frac{1}{2\beta}\left(s_*^{N_{\text{conv}}+1,\beta} \cdot \mathcal{F}\left(s_*^{N_{\text{conv}},\beta}\right)^\top - s_*^{N_{\text{conv}}+1,-\beta} \cdot \mathcal{F}\left(s_*^{N_{\text{conv}},-\beta}\right)^\top\right) \\ \forall n \in [1, N_{\text{conv}}-1]: \quad \Delta w_{n+1} = \frac{1}{2\beta}\left(\mathcal{P}^{-1}(s_*^{n+1,\beta}) \bullet s_*^{n,\beta} - \mathcal{P}^{-1}(s_*^{n+1,-\beta}) \bullet s_*^{n,-\beta}\right) \\ \Delta w_1 = \frac{1}{2\beta}\left(\mathcal{P}^{-1}(s_*^{1,\beta}) \bullet x - \mathcal{P}^{-1}(s_*^{1,-\beta}) \bullet x\right) \end{cases} . \tag{S18}$$

4.2.3 Implementation details in PyTorch.

The equation of the dynamics as well as the EP estimates computation can be expressed as derivatives of the primitive function Φ. Therefore, the automatic differentiation framework provided by PyTorch can be leveraged to implement implicitly the equations of the dynamics and the EP estimates computation by differentiating Φ. Although this implementation is slower than explicitly implementing the equations of the dynamics, it is more flexible in terms of network architecture as Φ is relatively easy to compute.

4.3 Convolutional RNNs with asymmetric connections

In this section, we write the explicit definition of the dynamics and the learning rule of a convolutional architecture with asymmetric connections where forward and backward connections are no longer constrained to be equal-valued. In this setting, we use the Cross-Entropy loss function along with a softmax readout to implement the output layer of the network.

4.3.0.1 Equations of the dynamics.

In this setting, the dynamics Eq. (S15) have simply to be changed into:

$$\begin{cases} s_{t+1}^{n+1} = \sigma\left(\mathcal{P}(w_{n+1}^{\text{f}} \star s_t^n) + \tilde{w}_{n+2}^{\text{b}} \star \mathcal{P}^{-1}(s_t^{n+2})\right), \qquad \forall n \in [0, N^{\text{conv}}-2] \\ s_{t+1}^{N^{\text{conv}}} = \sigma\left(\mathcal{P}(w_{N^{\text{conv}}}^{\text{f}} \star s_t^{N^{\text{conv}}-1}) + \mathcal{F}^{-1}({w_{N^{\text{conv}}+1}^{\text{b}}}^\top \cdot s_t^1)\right), \\ s_{t+1}^{N^{\text{conv}}+1} = \sigma\left(w_{N^{\text{conv}}+1}^{\text{f}} \cdot \mathcal{F}(s_t^{N^{\text{conv}}}) + {w_{N^{\text{conv}}+2}^{\text{b}}}^\top \cdot s_t^{N^{\text{conv}}+2}\right), \\ s_{t+1}^{n+1} = \sigma\left(w_{n+1}^{\text{f}} \cdot s_t^n + {w_{n+2}^{\text{b}}}^\top \cdot s_t^{n+2}\right), \qquad \forall n \in [N^{\text{conv}}+1, N^{\text{tot}}-3] \\ s_{t+1}^{N^{\text{tot}}-1} = \sigma\left(w_{N^{\text{tot}}-1}^{\text{f}} \cdot s_t^{N^{\text{tot}}-2}\right) + \beta {w_{\text{out}}}^\top \cdot (y - \hat{y}), \\ \hat{y} = \text{softmax}(w_{\text{out}} \cdot s_t^{N^{\text{tot}}-1}), \end{cases} \tag{S19}$$

where we distinguish now between forward and backward connections: $w_n^{\text{f}} \neq w_n^{\text{b}} \quad \forall n \in [1, N_{\text{tot}}-2]$.

4.3.0.2 Original Vector Field learning rule (VF).

The symmetric version of the original Vector Field learning rule is defined as:

$$\widehat{\nabla}_{\text{sym}}^{\text{VF}}(\beta) \triangleq \frac{1}{2\beta}\frac{\partial F}{\partial \theta}(x, s_*, \theta)^\top \cdot \left(s_*^{\beta} - s_*^{-\beta}\right), \tag{S20}$$

which yields in the case of softmax read-out:

$$
\begin{cases}
\Delta w_{\text{out}} = -\frac{1}{2}\left(\left(\widehat{y_*^\beta} - y\right) \cdot s_*^{\beta,N^\top} + \left(\widehat{y_*^{-\beta}} - y\right) \cdot s_*^{-\beta,N^\top}\right). \\
\forall n \in [N_{\text{conv}}+2, N_{\text{tot}}-2]: \quad \Delta w_n^{\text{f}} = \frac{1}{2\beta}\left(s_*^{n+1,\beta} - s_*^{n+1,-\beta}\right) \cdot s_*^{n^\top} \\
\forall n \in [N_{\text{conv}}+2, N_{\text{tot}}-2]: \quad \Delta w_n^{\text{b}} = \frac{1}{2\beta} s_*^{n+1} \cdot \left(s_*^{n,\beta} - s_*^{n,-\beta}\right)^\top \\
\Delta w_{N_{\text{conv}}+1}^{\text{f}} = \frac{1}{2\beta}\left(s_*^{N_{\text{conv}}+1,\beta} - s_*^{N_{\text{conv}}+1,-\beta}\right) \cdot \mathcal{F}\left(s_*^{N_{\text{conv}}}\right)^\top \\
\Delta w_{N_{\text{conv}}+1}^{\text{b}} = \frac{1}{2\beta} s_*^{N_{\text{conv}}+1} \cdot \left(\mathcal{F}\left(s_*^{N_{\text{conv}},\beta}\right) - \mathcal{F}\left(s_*^{N_{\text{conv}},-\beta}\right)\right)^\top \\
\forall n \in [1, N_{\text{conv}}-1]: \quad \Delta w_{n+1}^{\text{f}} = \frac{1}{2\beta}\left(\mathcal{P}^{-1}(s_*^{n+1,\beta}) - \mathcal{P}^{-1}(s_*^{n+1,-\beta})\right) \bullet s_*^n \\
\forall n \in [1, N_{\text{conv}}-1]: \quad \Delta w_{n+1}^{\text{b}} = \frac{1}{2\beta}\mathcal{P}^{-1}(s_*^{n+1}) \bullet \left(s_*^{n,\beta} - s_*^{n,-\beta}\right) \\
\Delta w_1 = \frac{1}{2\beta}\left(\mathcal{P}^{-1}(s_*^{1,\beta}) \bullet x - \mathcal{P}^{-1}(s_*^{1,-\beta}) \bullet x\right)
\end{cases}. \tag{S21}
$$

Importantly, note that $\Delta w_n^{\text{f}} \neq \Delta w_n^{\text{b}} \quad \forall n \in [1, N_{\text{tot}}-2]$.

4.3.0.3 Kolen-Pollack algorithm.

When forward and backward weights have a common gradient estimate, and a weight decay term λ, they converge to the same values. We recall the proof, noting t the iteration step and taking the notations of section 3.3, the update rule follows:

$$
\begin{cases}
\theta_{\text{f}}(t+1) = \theta_{\text{f}}(t) + \Delta\theta_{\text{f}} \\
\theta_{\text{b}}(t+1) = \theta_{\text{b}}(t) + \Delta\theta_{\text{b}}
\end{cases}.
$$

We can then write

$$
\begin{aligned}
\theta_{\text{f}}(t+1) - \theta_{\text{b}}(t+1) &= \theta_{\text{f}}(t) - \theta_{\text{b}}(t) + \Delta\theta_{\text{f}} - \Delta\theta_{\text{b}} \\
&= \theta_{\text{f}}(t) - \theta_{\text{b}}(t) - \eta\lambda\left(\theta_{\text{f}}(t) - \theta_{\text{b}}(t)\right) \\
&= (1-\eta\lambda)\left(\theta_{\text{f}}(t) - \theta_{\text{b}}(t)\right),
\end{aligned}
$$

where we use the fact that the estimates are the same for both parameters, such that they cancel out. Then by recursion :

$$
\theta_{\text{f}}(t) - \theta_{\text{b}}(t) = (1-\eta\lambda)^t\left(\theta_{\text{f}}(0) - \theta_{\text{b}}(0)\right) \underset{t\to\infty}{\to} 0, \quad \text{since} \quad |1-\eta\lambda| < 1.
$$

4.3.0.4 Kolen-Pollack Vector Field learning rule (KP-VF).

We remind here that the new learning rule proposed in this paper to train convNets with asymmetric connections is defined as:

$$\left\{\begin{array}{l}\Delta\theta_{\rm f} = \eta\left(\widehat{\nabla}^{\rm KP-VF}_{\rm sym}(\beta) - \lambda\theta_{\rm f}\right)\\ \Delta\theta_{\rm b} = \eta\left(\widehat{\nabla}^{\rm KP-VF}_{\rm sym}(\beta) - \lambda\theta_{\rm b}\right)\end{array}\right., \quad \text{with} \quad \widehat{\nabla}^{\rm KP-VF}_{\rm sym}(\beta) = \frac{1}{2}(\overline{\nabla^{\rm VF}_{\theta_{\rm f}}}(\beta) + \overline{\nabla^{\rm VF}_{\theta_{\rm b}}}(\beta)), \tag{S22}$$

where:

$$\forall \mathrm{i} \in \{\mathrm{f}, \mathrm{b}\}, \qquad \overline{\nabla^{\rm VF}_{\theta_{\rm i}}}(\beta) = \frac{1}{2\beta}\left(\frac{\partial F}{\partial\theta_{\rm i}}^{\top}(x, s_*^{\beta}, \theta)\cdot s_*^{\beta} - \frac{\partial F}{\partial\theta_{\rm i}}^{\top}(x, s_*^{-\beta}, \theta)\cdot s_*^{-\beta}\right). \tag{S23}$$

More specifically, applying Eq. (S23) to Eq. (S19) yields:

$$\left\{\begin{array}{l}
\forall n \in [N_{\rm conv}+2, N_{\rm tot}-2]: \\
\qquad \overline{\nabla^{\rm VF}_{w_n^{\rm f}}}(\beta) = \overline{\nabla^{\rm VF}_{w_n^{\rm b}}}(\beta) = \frac{1}{2\beta}\left(s_*^{n+1,\beta}\cdot {s_*^{n,\beta}}^{\top} - s_*^{n+1,-\beta}\cdot {s_*^{n,-\beta}}^{\top}\right) \\
\overline{\nabla^{\rm VF}_{w^{\rm f}_{N_{\rm conv}+1}}}(\beta) = \overline{\nabla^{\rm VF}_{w^{\rm b}_{N_{\rm conv}+1}}}(\beta) = \\
\qquad \frac{1}{2\beta}\left(s_*^{N_{\rm conv}+1,\beta}\cdot \mathcal{F}\left(s_*^{N_{\rm conv},\beta}\right)^{\top} - s_*^{N_{\rm conv}+1,-\beta}\cdot\mathcal{F}\left(s_*^{N_{\rm conv},-\beta}\right)^{\top}\right) \\
\forall n \in [1, N_{\rm conv}-1]: \\
\overline{\nabla^{\rm VF}_{w^{\rm f}_{n+1}}}(\beta) = \frac{1}{2\beta}\left(\mathcal{P}^{-1}\left(s_*^{n+1,\beta}, \mathrm{ind}_{\mathcal{P}}\left(w^{\rm f}_{n+1}\star s_*^{n,\beta}\right)\right)\bullet s_*^{n,\beta}\right. \\
\qquad\qquad \left.-\mathcal{P}^{-1}\left(s_*^{n+1,-\beta}, \mathrm{ind}_{\mathcal{P}}\left(w^{\rm f}_{n+1}\star s_*^{n,-\beta}\right)\right)\bullet s_*^{n,-\beta}\right) \\
\forall n \in [1, N_{\rm conv}-1]: \\
\overline{\nabla^{\rm VF}_{w^{\rm b}_{n+1}}}(\beta) = \frac{1}{2\beta}\left(\mathcal{P}^{-1}\left(s_*^{n+1,\beta}, \mathrm{ind}_{\mathcal{P}}\left(w^{\rm b}_{n+1}\star s_*^{n,\beta}\right)\right)\bullet s_*^{n,\beta}\right. \\
\qquad\qquad \left.-\mathcal{P}^{-1}\left(s_*^{n+1,-\beta}, \mathrm{ind}_{\mathcal{P}}\left(w^{\rm b}_{n+1}\star s_*^{n,-\beta}\right)\right)\bullet s_*^{n,-\beta}\right)
\end{array}\right. . \tag{S24}$$

Combining Eqs. (S24) with Eq. (S22) gives the associated parameter updates. The updates for w_1 and $w_{\rm out}$ are the same than those of Eq. (S21). Importantly, note that while $\forall n \in [N_{\rm conv}+1, N_{\rm tot}-2]$: $\overline{\nabla^{\rm VF}_{w_n^{\rm f}}}(\beta) = \overline{\nabla^{\rm VF}_{w_n^{\rm b}}}(\beta)$, we have $\forall n \in [1, N_{\rm conv}-1]$: $\overline{\nabla^{\rm VF}_{w_n^{\rm f}}}(\beta) \neq \overline{\nabla^{\rm VF}_{w_n^{\rm b}}}(\beta)$ because of inverse pooling. In other words, the updates of the convolutional filters do not solely depend on the pre and post synaptic activations but also on the location of the maximal elements within each pooling window, itself depending on the filter considered. Hence the motivation to average $\overline{\nabla^{\rm VF}_{w_n^{\rm f}}}(\beta)$ and $\overline{\nabla^{\rm VF}_{w_n^{\rm b}}}(\beta)$ and use this quantity to update to $w_n^{\rm b}$ and $w_n^{\rm f}$ and apply the Kolen-Pollack technique.

4.3.0.5 Implementation details in PyTorch.

The dynamics in the case of asymmetric connections does not derive from a primitive function Φ. Therefore, it is not possible to implicitly get the dynamics by differentiating one primitive function. A way around is to get the asymmetric dynamics by differentiating one quantity $\tilde{\Phi}^n$ by layer. This quantity is not a primitive function and is especially designed to get the right equations once differentiated. We define $\tilde{\Phi}^n(w_n^{\rm f}, w_{n+1}^{\rm b}, s^{n-1}, s^n)$ by:

$$\begin{cases} \forall n \in [1, N_{\rm conv} - 1] : \tilde{\Phi}^n = s^n \bullet \mathcal{P}(w_n^{\rm f} \star s^{n-1}) + s^{n+1} \bullet \mathcal{P}(w_{n+1}^{\rm b} \star s^n) \\ \tilde{\Phi}^{N_{\rm conv}} = s^{N_{\rm conv}} \bullet \mathcal{P}(w_{N_{\rm conv}}^{\rm f} \star s^{N_{\rm conv}-1}) + s^{N_{\rm conv}+1} \cdot w_{N_{\rm conv}+1}^{\rm b} \cdot \mathcal{F}(s^{N_{\rm conv}}) \\ \forall n \in [N_{\rm conv} + 1, N_{\rm tot} - 1] : \tilde{\Phi}^n = s^n \cdot w_n^{\rm f} \cdot s^{n-1} + s^{n+1} \cdot w_{n+1}^{\rm b} \cdot s^n \\ \tilde{\Phi}^{N_{\rm tot}-1} = s^{N_{\rm tot}-1} \cdot w_{N_{\rm tot}-1}^{\rm f} \cdot s^{N_{\rm tot}-2} + \beta \ell(s^{N_{\rm tot}-1}, y, w_{\rm out}) \end{cases}, \tag{S25}$$

where ℓ is defined by Eq. (18), and $\beta = 0$ in the first phase. Then, $\forall n \in [1, N^{\rm tot} - 1]$, the dynamics of Eq. (S19) read:

$$s_{t+1}^n = \sigma\left(\frac{\partial \tilde{\Phi}^n}{\partial s^n}(w_n^{\rm f}, w_{n+1}^{\rm b}, s_t^{n-1}, s_t^n)\right). \tag{S26}$$

The original VF update of Eq. (S21) can be written as $\forall n \in [1, N^{\rm tot} - 1], \forall {\rm i} \in \{{\rm f}, {\rm b}\}$:

$$\Delta w_n^{\rm i} = \frac{1}{2\beta}\left(\frac{\partial \tilde{\Phi}^n}{\partial w_n^{\rm i}}(s_*^{n,\beta}, s_*^{n-1}) - \frac{\partial \tilde{\Phi}^n}{\partial w_n^{\rm i}}(s_*^{n,-\beta}, s_*^{n-1})\right), \tag{S27}$$

and Eq. (S24) as:

$$\overline{\nabla}_{w_n^{\rm i}}^{\rm VF}(\beta) = \frac{1}{2\beta}\left(\frac{\partial \tilde{\Phi}^n}{\partial w_n^{\rm i}}(s_*^{n,\beta}, s_*^{n-1,\beta}) - \frac{\partial \tilde{\Phi}^n}{\partial w_n^{\rm i}}(s_*^{n,-\beta}, s_*^{n-1,-\beta})\right). \tag{S28}$$

4.4 Random-sign estimate variance

The results presented in Table 1 consists of five runs. In the case of the EP random-sign estimate, one run among the five collapses to random guess similar to the one-sided estimate. In order to test the frequency of such a phenomenon, we performed another five runs with both symmetric and random-sign estimates. The results for each run presented in Table S1 show that two trials among ten are unstable, confirming further the high variance nature of the random-sign estimate.

Table S1. Best test error comparison between random-sign and symmetric estimates, for ten runs.

Run index	EP random-sign	EP symmetric
1	12.97	12.24
2	12.72	12.31
3	12.30	12.68
4	12.45	12.43
5	12.78	12.57
6	12.66	12.55
7	12.84	12.44
8	12.59	12.52
9	57.32	12.85
10	89.98	12.60
Mean	24.86	**12.52**
w/o collapse	**12.66**	N.A

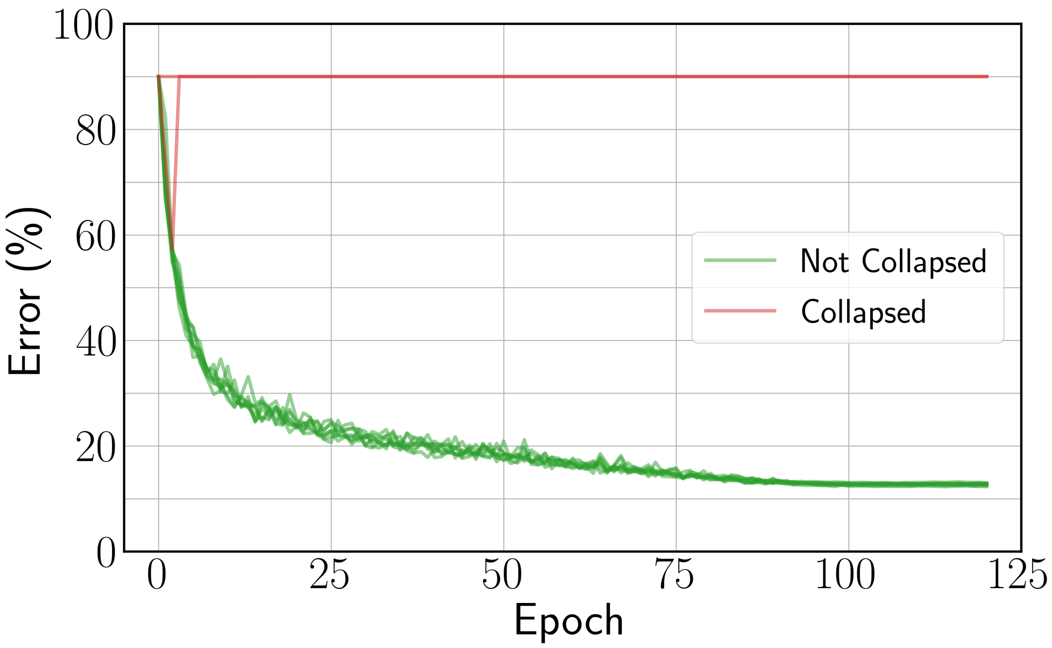

Figure S1. Test error curve of each run with the Squared Error loss function and random-sign estimate. The two collapsed runs among the ten trials are steady to 90% because in such cases the network typically outputs the same class for each data point.

4.5 Adding dropout

We adapt dropout (Srivastava et al., 2014) for convergent RNNs by shutting some units to zero with probability $p < 1$ when computing $\Phi(x, s_t, \theta)$. We multiply the remaining active units by the factor $\frac{1}{1-p}$ to keep the same neural activity on average, so that the learning rule is rescaled by $\left(\frac{1}{1-p}\right)^2$. The dropped out units are the same within one training iteration but they differ across the examples of one mini batch. In our experiments we use $p = 0.1$ on the last convolutional layer before the linear classifier. The results are reported in Table 1.

4.6 Changing the activation function

Previous implementations of EP used a shifted hard sigmoid activation function:

$$\sigma(x) = \max(0, \min(x, 1)). \tag{S29}$$

In their experiments with ConvNets on MNIST, Ernoult et al. (2019) observed saturating units that cannot pass error signals during the second phase. In this work, to mitigate this effect, we have rescaled by a factor $1/2$ the slope of the activation function to ease signal propagation and prevent saturation, therefore changing Eq. (S29) into:

$$\sigma(x) = \max\left(0, \min\left(\frac{x}{2}, 1\right)\right). \tag{S30}$$

5 WEIGHT ALIGNMENT FOR ASYMMETRIC CONNECTIONS

The angle α between forward and backward weights is defined as :

$$\alpha = \frac{180}{\pi}\mathrm{Acos}\left(\frac{w^{\mathrm{b}} \bullet w^{\mathrm{f}}}{\|w^{\mathrm{b}}\|\|w^{\mathrm{f}}\|}\right), \quad \text{where} \quad \|w\| = \sqrt{w \bullet w}. \tag{S31}$$

Fig. 5 shows the angle between forward and backward weights during training on CIFAR-10 for both the original VF learning rule (dashed) and the new learning rule inspired by Kolen and Pollack (1994).

6 LAYER-WISE COMPARISON OF EP ESTIMATES

In this section we show on Fig. S2 more instances of Fig. 2 for each layer of the convolutional architecture.

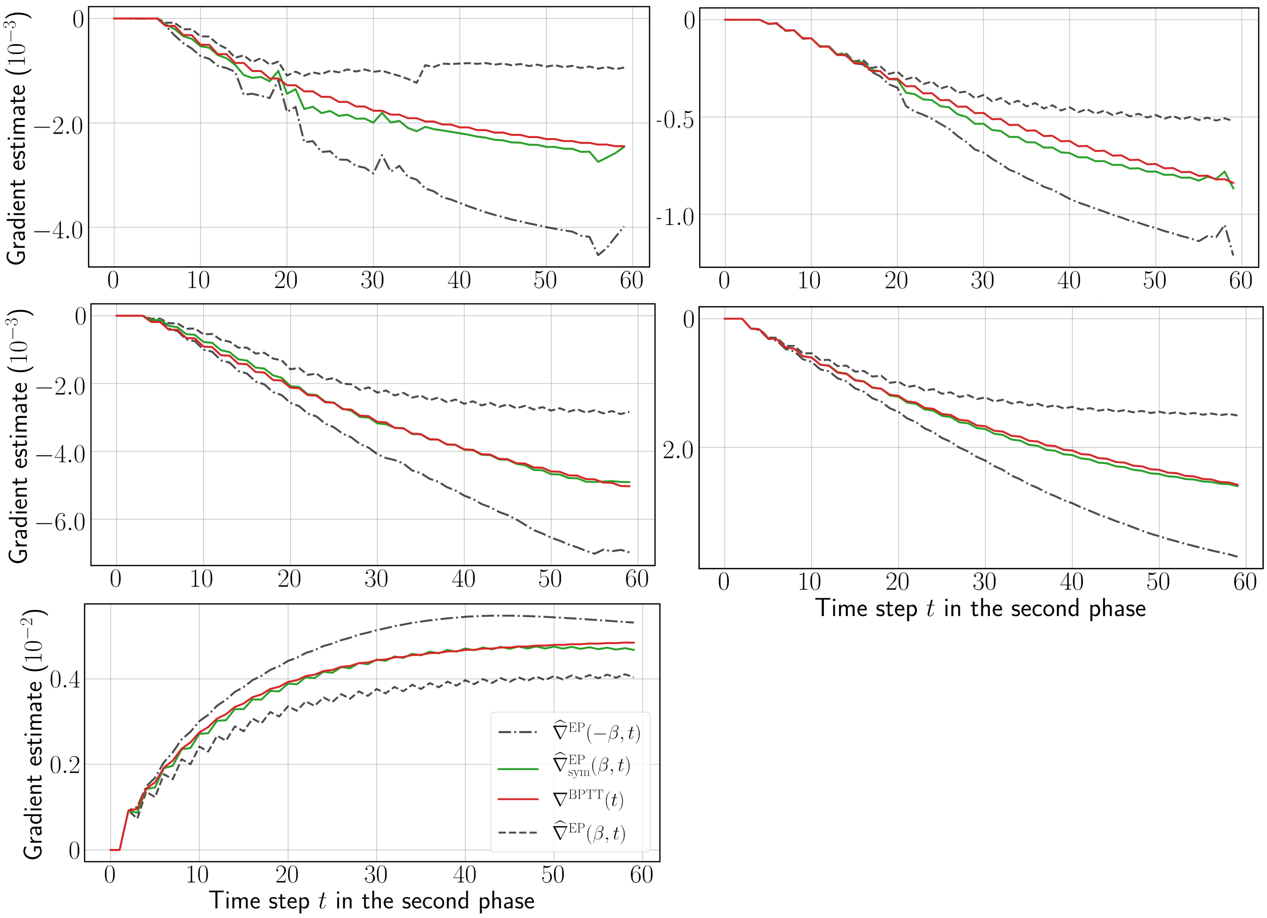

Figure S2. Layer-wise comparison between EP gradient estimates and BPTT gradients for 5 layers deep CNN on CIFAR-10 Data. Layer index increases from top to bottom, left to right, top-left being the first layer.

REFERENCES

Ernoult, M., Grollier, J., Querlioz, D., Bengio, Y., and Scellier, B. (2019). Updates of equilibrium prop match gradients of backprop through time in an rnn with static input. In *Advances in Neural Information Processing Systems*. 7081–7091

Ernoult, M., Grollier, J., Querlioz, D., Bengio, Y., and Scellier, B. (2020). Equilibrium propagation with continual weight updates. *arXiv preprint arXiv:2005.04168*

Kolen, J. F. and Pollack, J. B. (1994). Backpropagation without weight transport. In *Proceedings of 1994 IEEE International Conference on Neural Networks (ICNN'94)* (IEEE), vol. 3, 1375–1380

Scellier, B. and Bengio, Y. (2017). Equilibrium propagation: Bridging the gap between energy-based models and backpropagation. *Frontiers in computational neuroscience* 11, 24

Srivastava, N., Hinton, G., Krizhevsky, A., Sutskever, I., and Salakhutdinov, R. (2014). Dropout: a simple way to prevent neural networks from overfitting. *The journal of machine learning research* 15, 1929–1958
